# Supplementary material for: Supramolecular Amphiphiles Based on Pillar[5]arene and Meroterpenoids: Synthesis, Self-Association and Interaction with Floxuridine
Source: Int J Mol Sci. 2021 Jul 26;22(15):7950. doi: 10.3390/ijms22157950 (PMC8348429; doi:10.3390/ijms22157950)
Supplement: Supplementary file 1 [file ijms-22-07950-s001.zip › ijms-1307960-supplementary.pdf]

Supplementary Information

For

Supramolecular amphiphiles based on pillar[5]arene and meroterpenoids: synthesis, self-association and interaction with floxuridine

Alan A. Akhmedov, Dmitriy N. Shurpik, Pavel L. Padnya, Alena I. Khadieva, Rustem R. Gamirov, Yulia V. Panina, Asiya F. Gazizova, Denis Yu. Grishaev, Vitaliy V. Plemenkov and Ivan I. Stoikov.

Content

Figures S1-S12. ^{1}H NMR spectra of the compounds **2a-d, 3a-d, 4a-d** 2-7

Figures S13-S24. ^{13}C NMR spectra of the compounds **2a-d, 3a-d, 4a-d** 8-13

Figures S25-S36. FT-IR spectra of the compounds **2a-d, 3a-d, 4a-d** 14-19

Figures S37-S40. HRMS spectra of the compounds **3c,d, 4c,d** 20-21

Figures S41-42. Bindfit for **3a/5, 3b/5** (Fit data to 1:1, 1:2 and 2:1 Host-Guest equilibria) 22-23

Figures S43. UV-vis titration spectra for the system **3a/5, 3b/5** 24

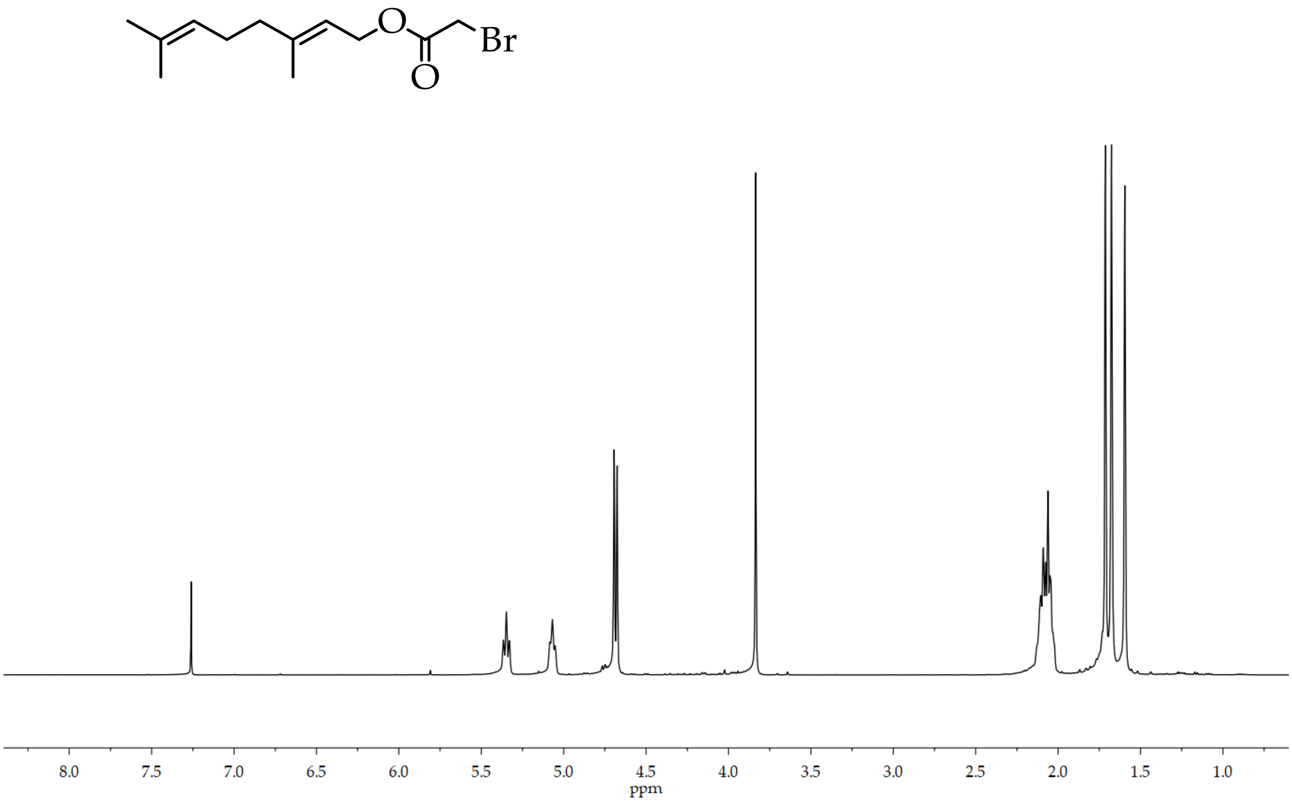

Figure S1. ^{1}H NMR spectrum of the compound **2a**, $CDCl_3$, 298 K, 400 MHz.

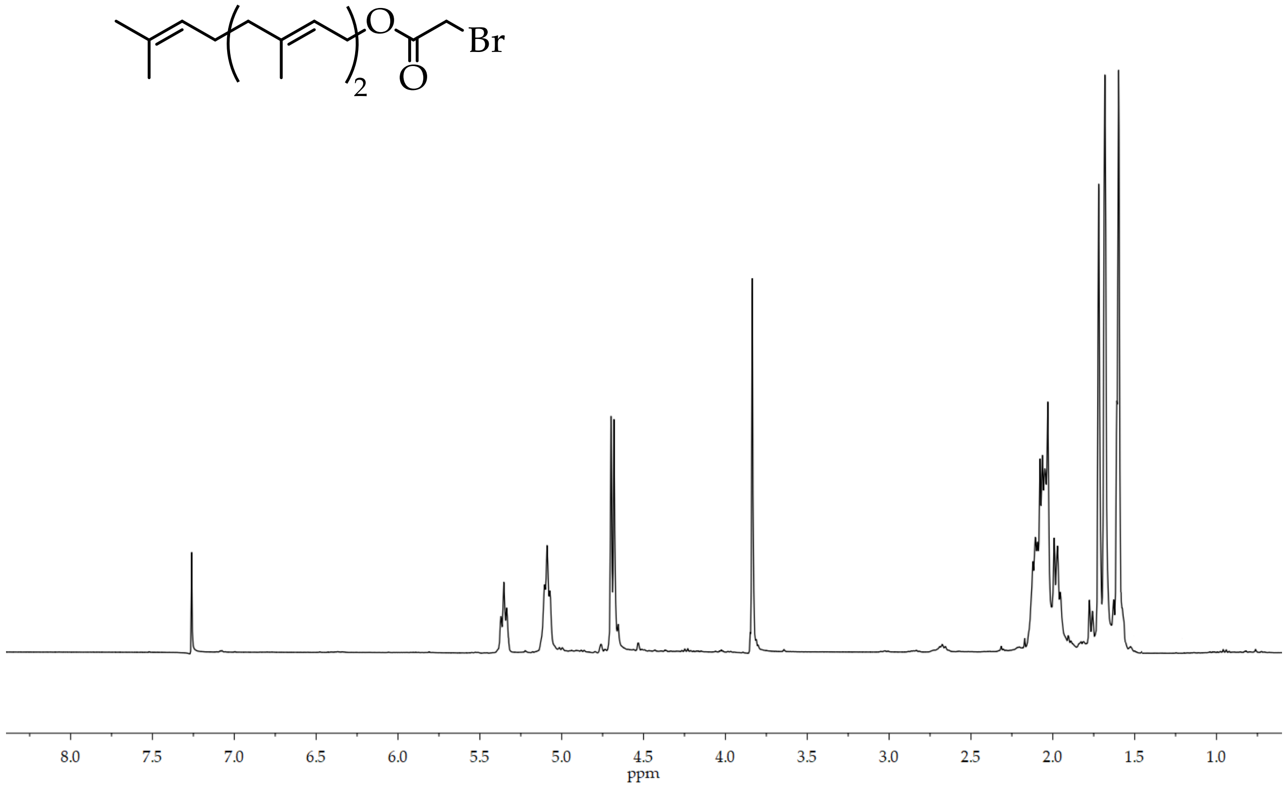

Figure S2. ^{1}H NMR spectrum of the compound **2b**, $CDCl_3$, 298 K, 400 MHz.

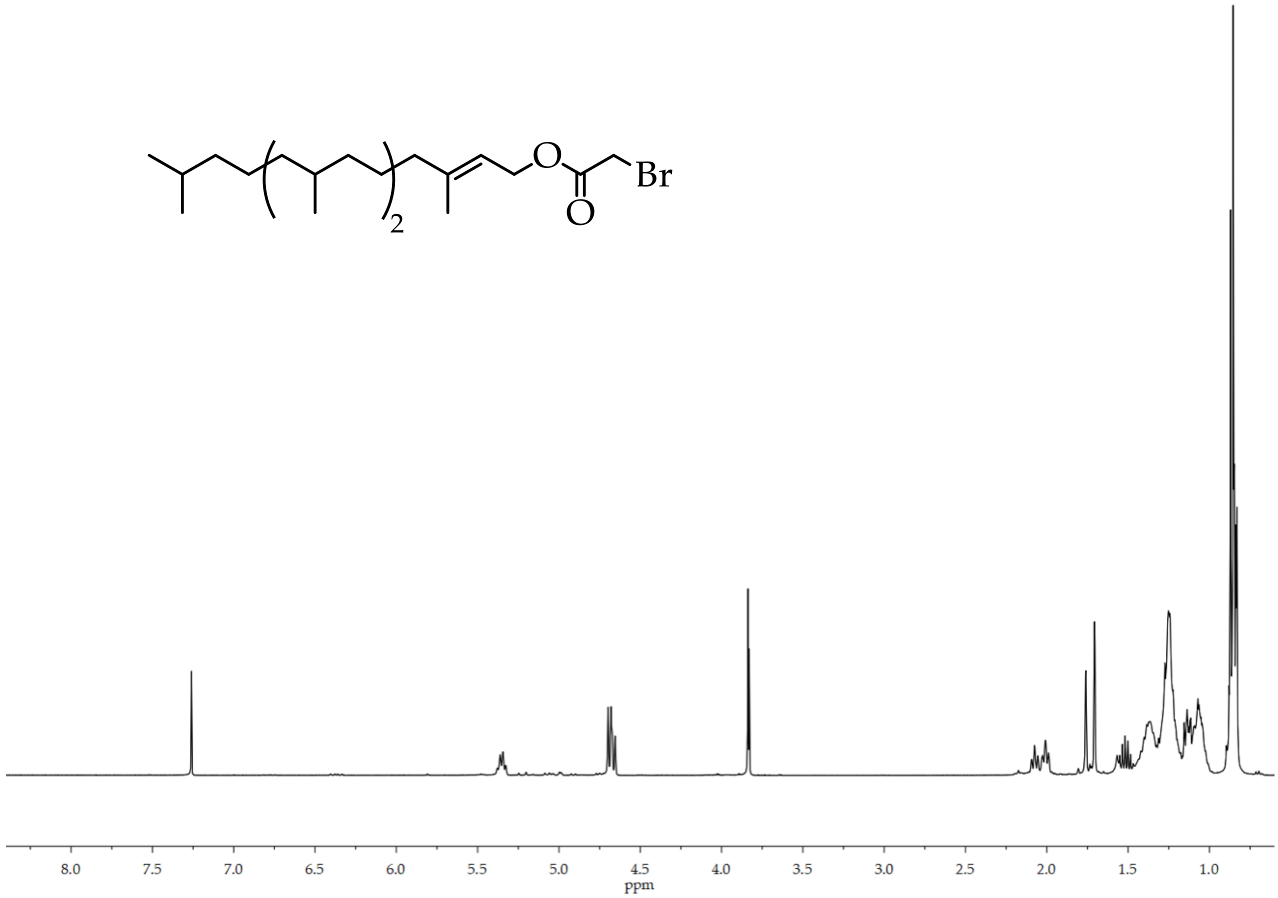

Figure S3. ^{1}H NMR spectrum of the compound **2c**, $CDCl_3$, 298 K, 400 MHz.

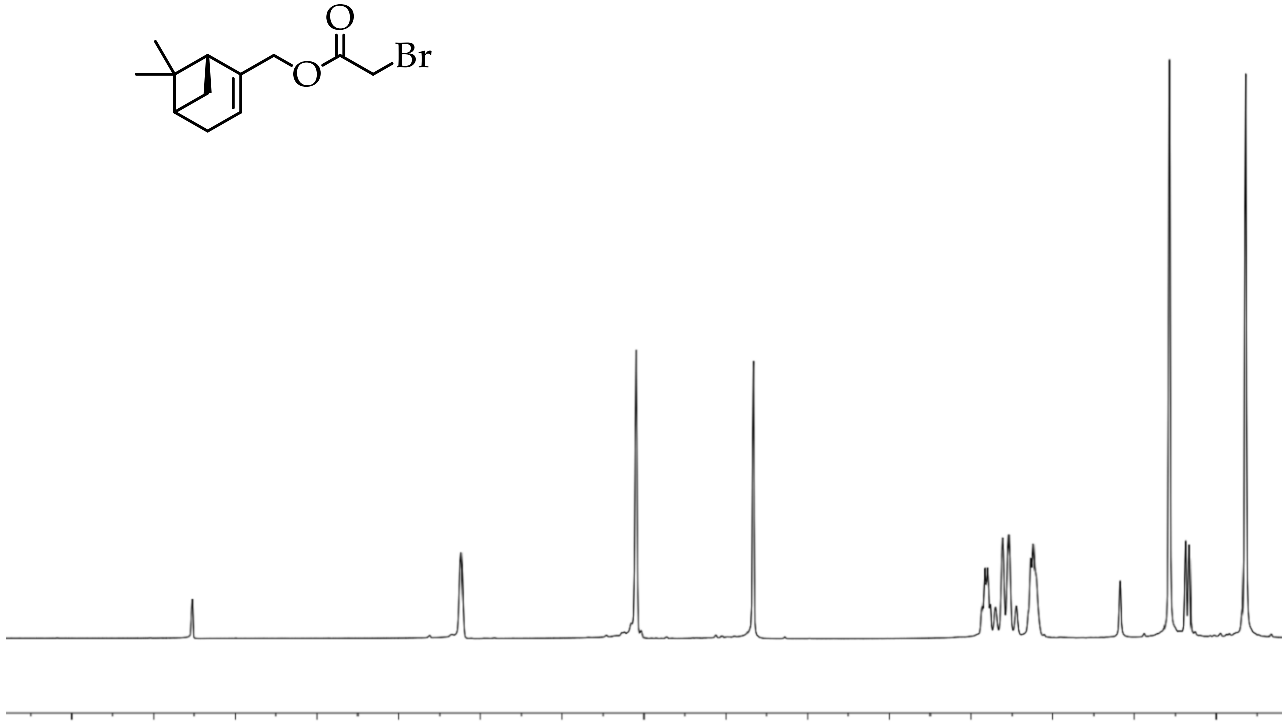

Figure S4. ^{1}H NMR spectrum of the compound **2d**, $CDCl_3$, 298 K, 400 MHz.

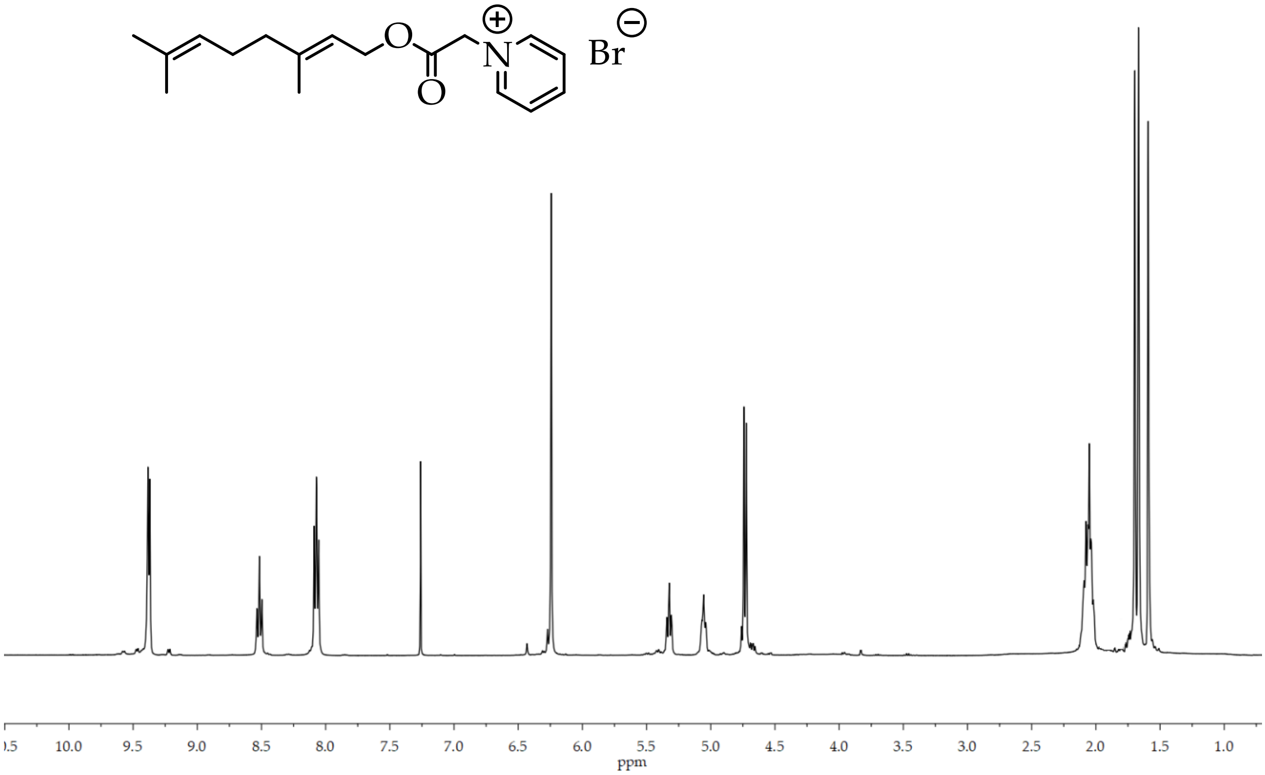

Figure S5. ^{1}H NMR spectrum of the compound **3a**, $CDCl_3$, 298 K, 400 MHz.

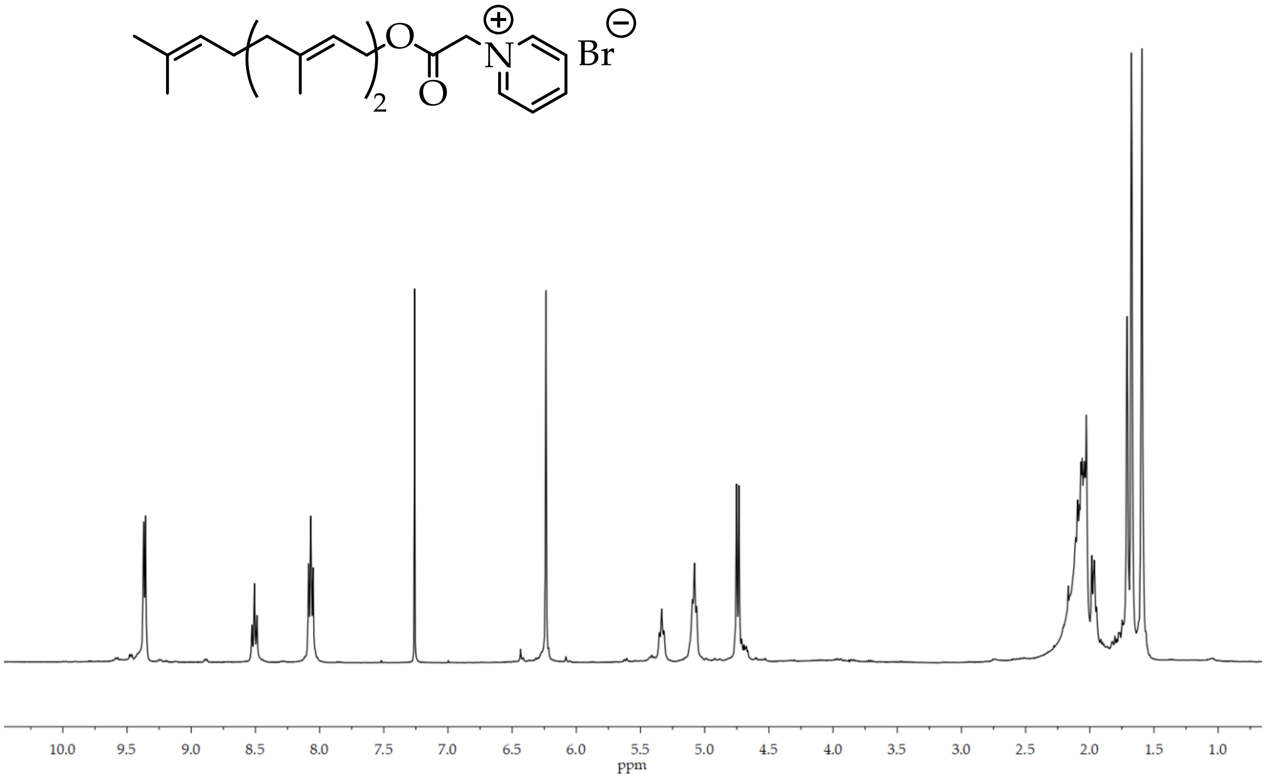

Figure S6. ^{1}H NMR spectrum of the compound **3b**, $CDCl_3$, 298 K, 400 MHz.

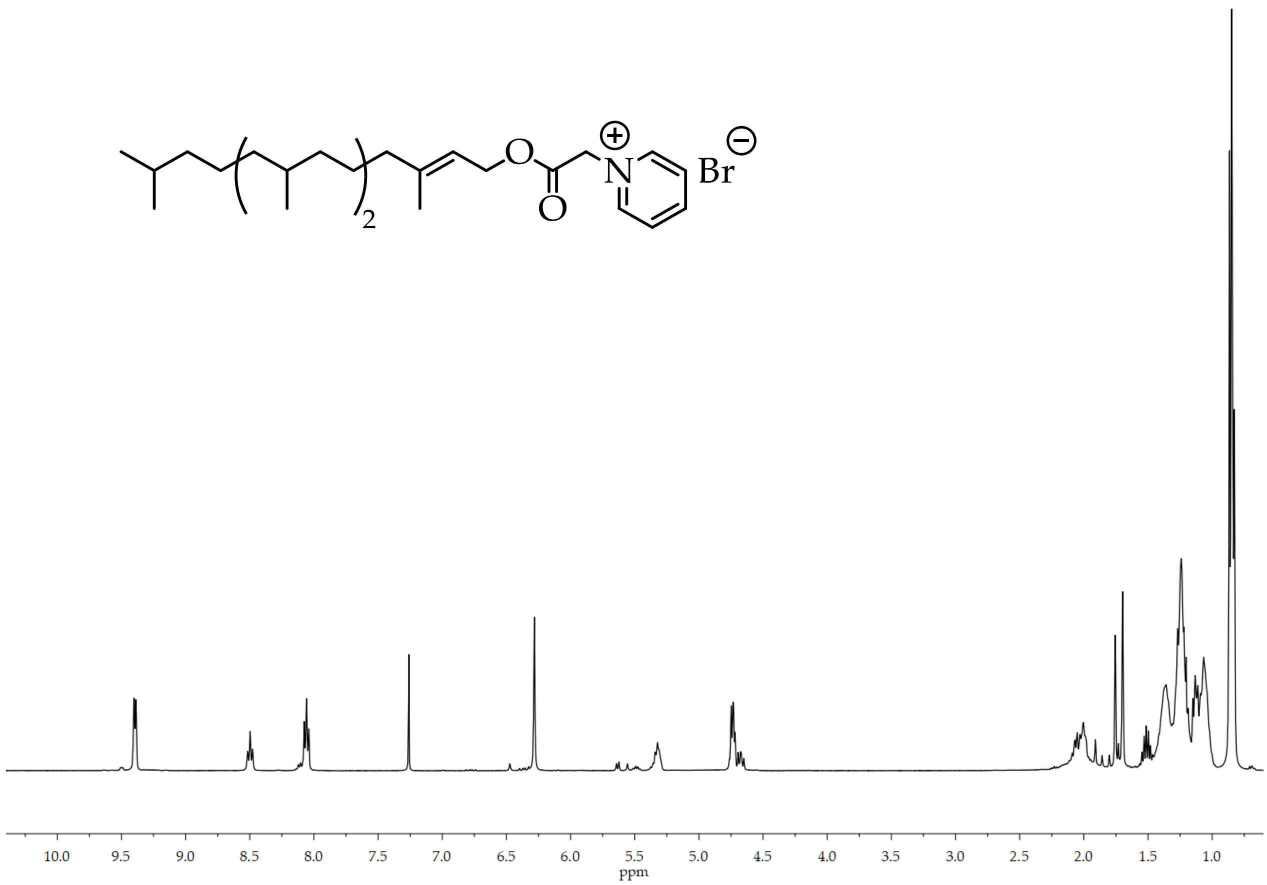

Figure S7. ^{1}H NMR spectrum of the compound **3c**, $CDCl_3$, 298 K, 400 MHz.

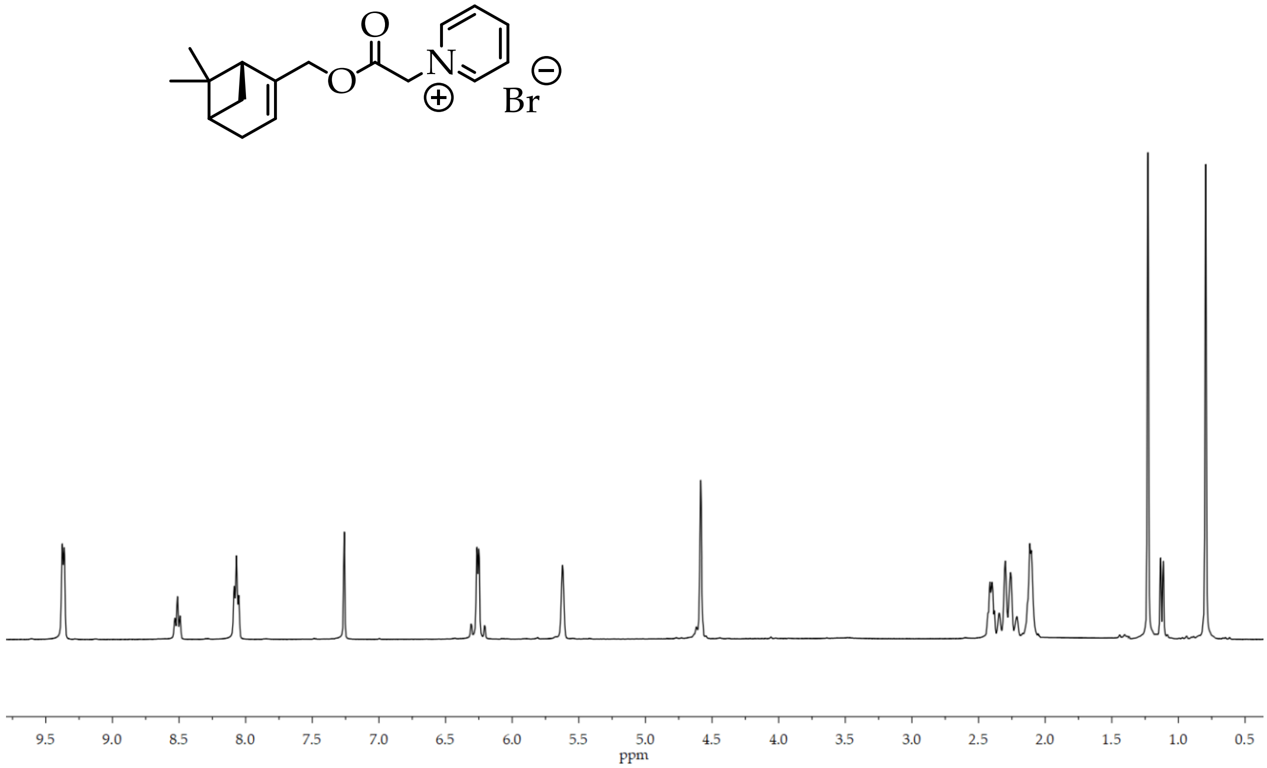

Figure S8. ^{1}H NMR spectrum of the compound **3d**, $CDCl_3$, 298 K, 400 MHz.

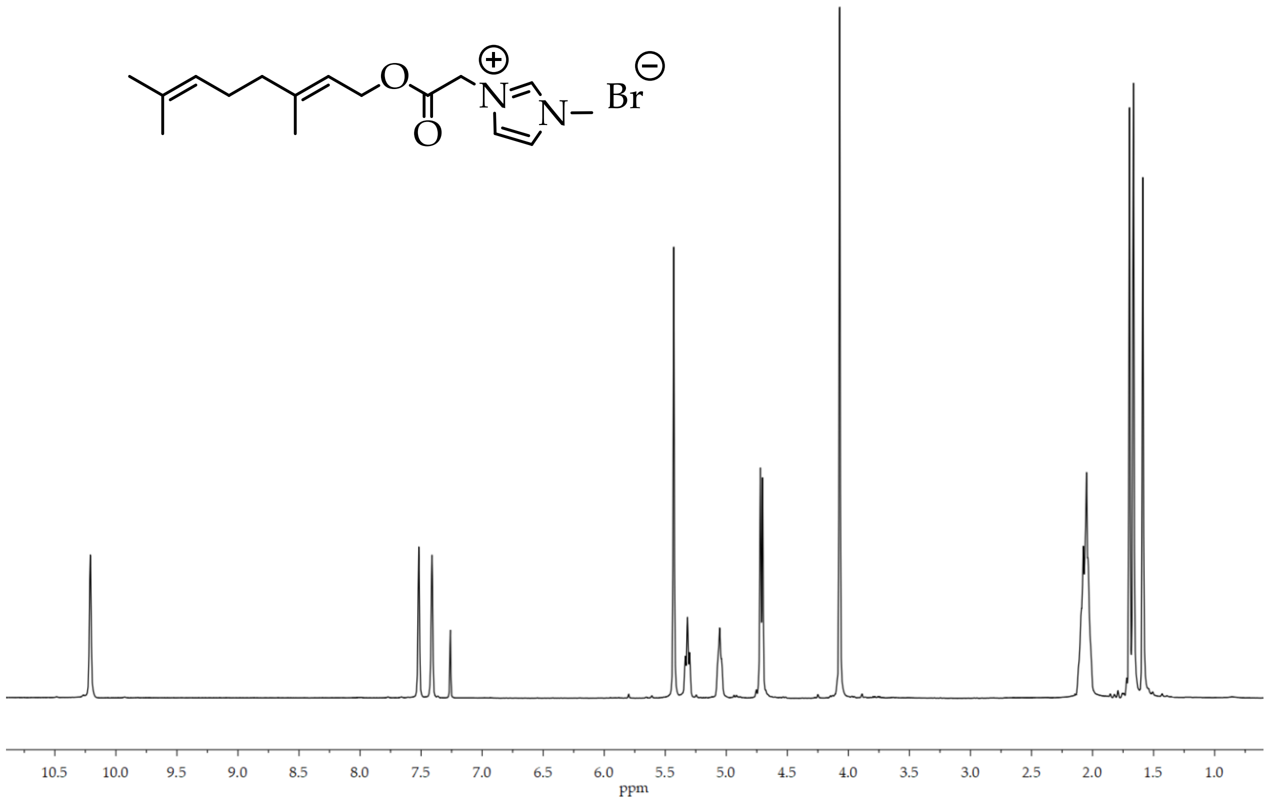

Figure S9. ¹H NMR spectrum of the compound **4a**, $CDCl_3$, 298 K, 400 MHz.

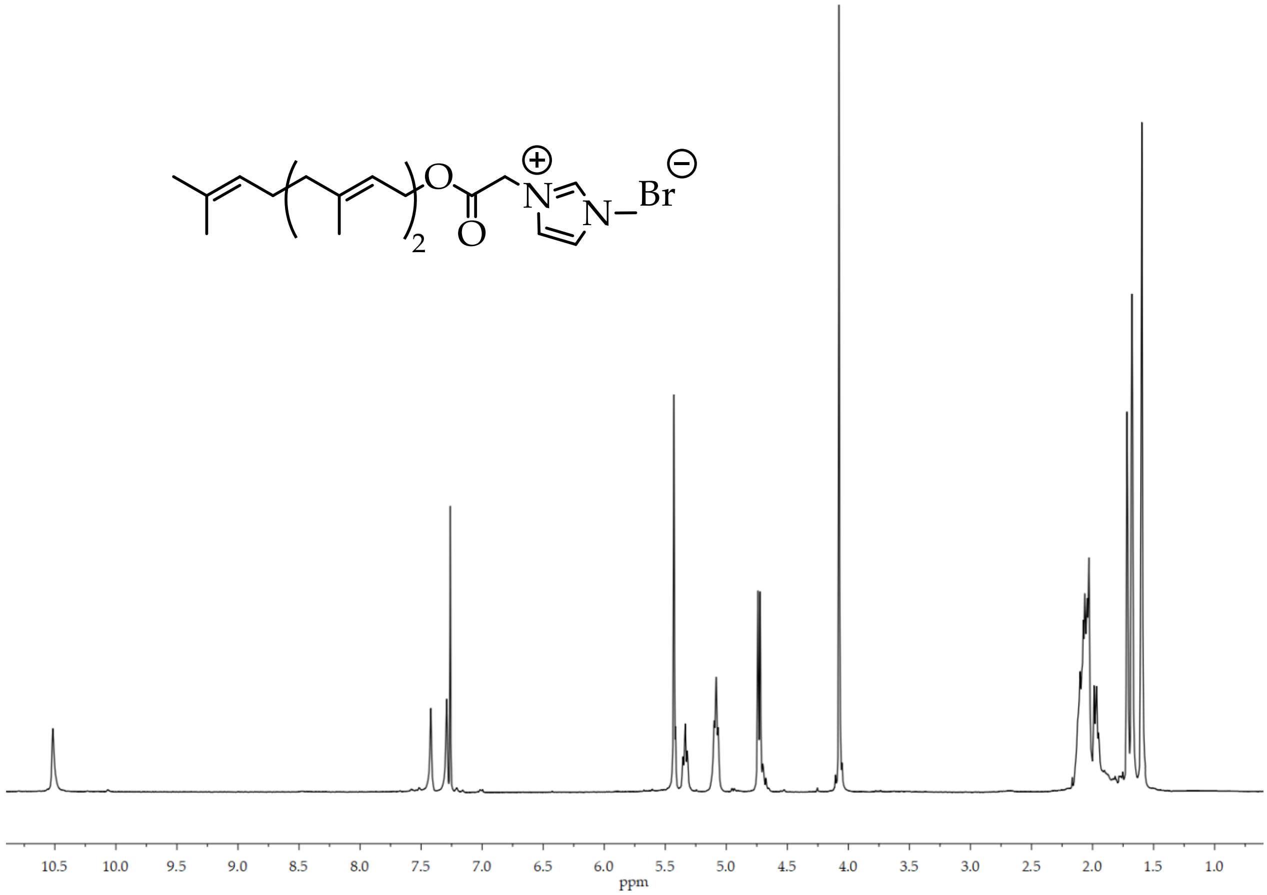

Figure S10. ¹H NMR spectrum of the compound **4b**, $CDCl_3$, 298 K, 400 MHz.

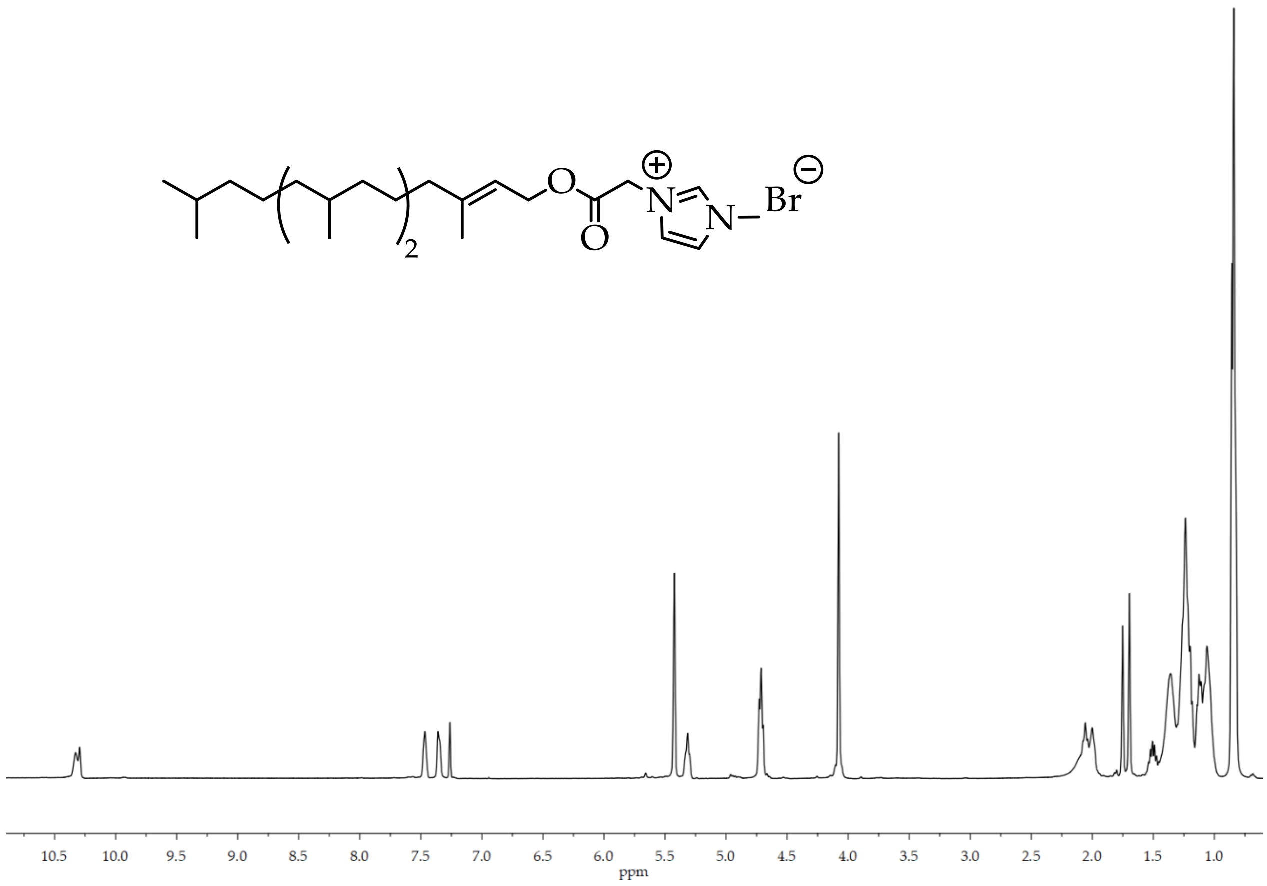

Figure S11. ^{1}H NMR spectrum of the compound **4c**, $CDCl_3$, 298 K, 400 MHz.

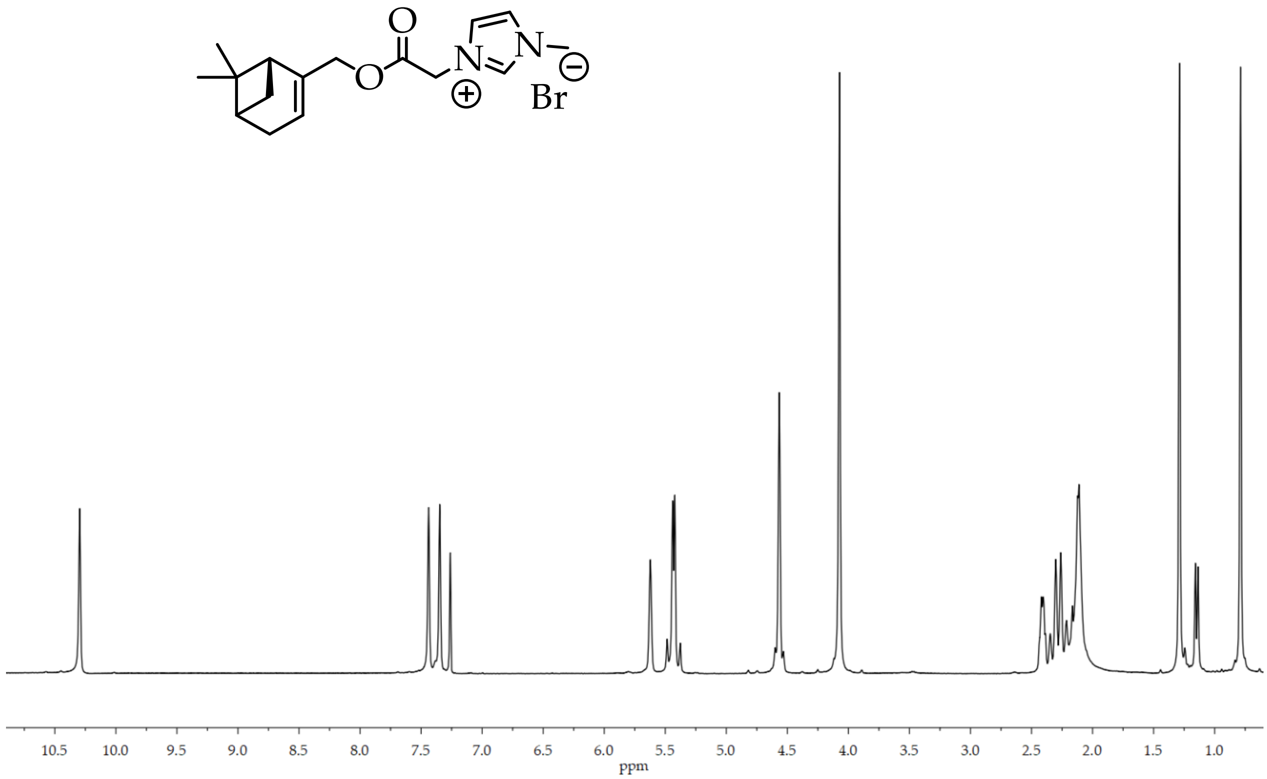

Figure S12. ^{1}H NMR spectrum of the compound **4d**, $CDCl_3$, 298 K, 400 MHz.

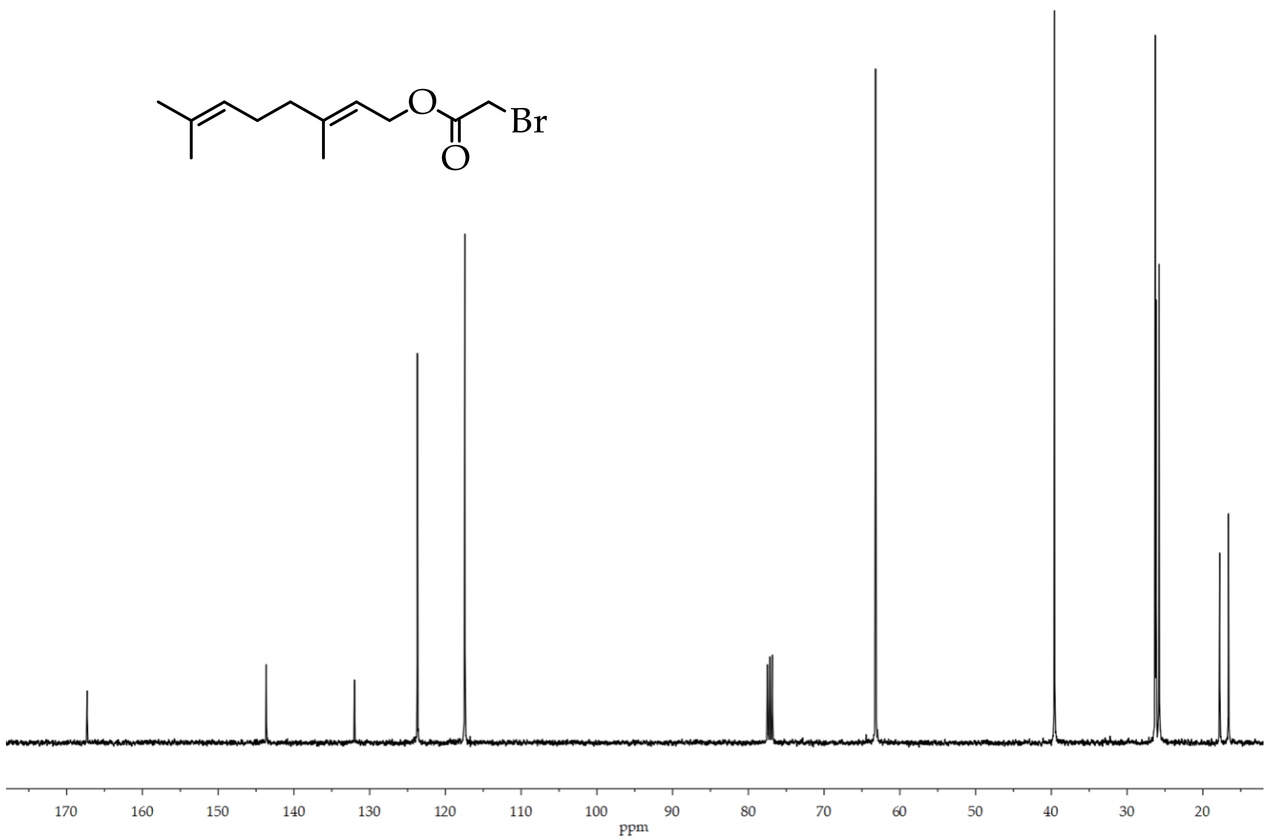

Figure S13. ^{13}C NMR spectrum of the compound **2a**, $CDCl_3$, 298 K, 100 MHz.

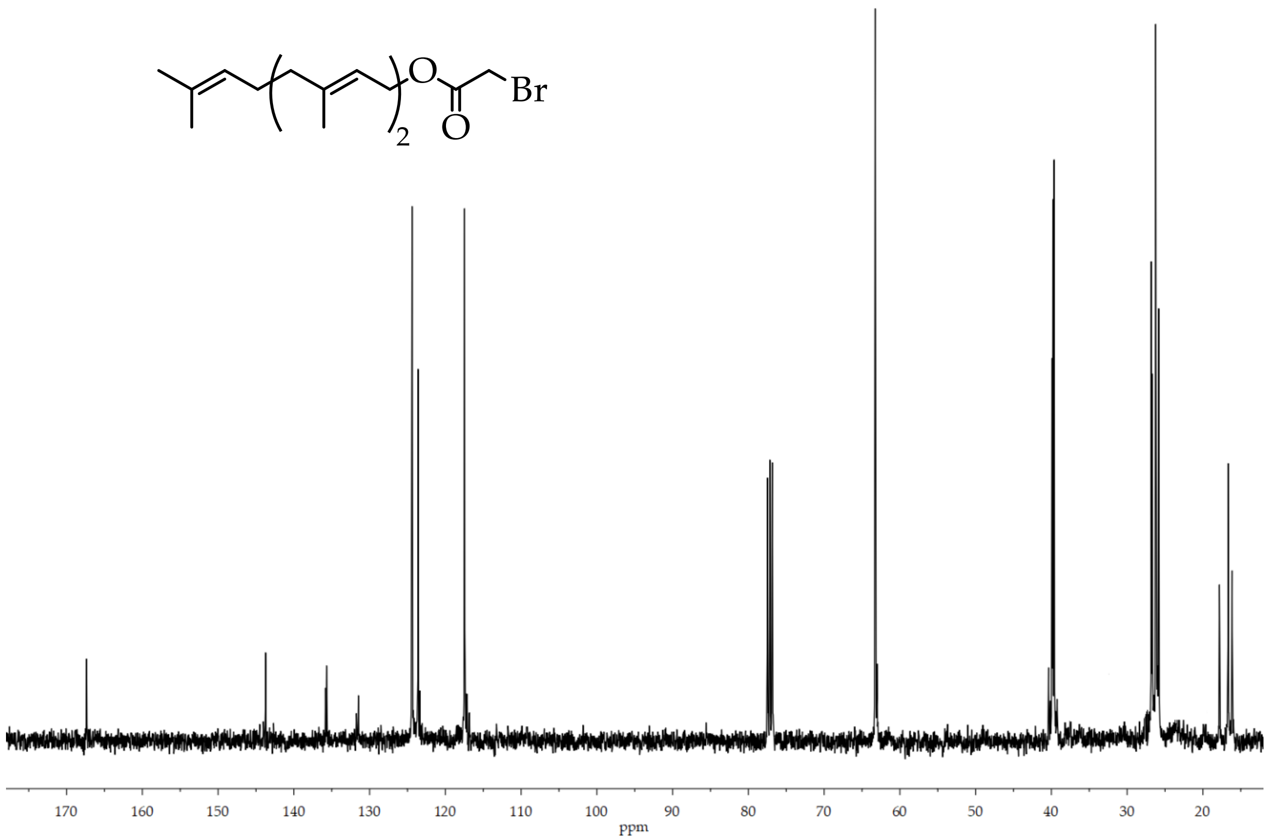

Figure S14. ^{13}C NMR spectrum of the compound **2b**, $CDCl_3$, 298 K, 100 MHz.

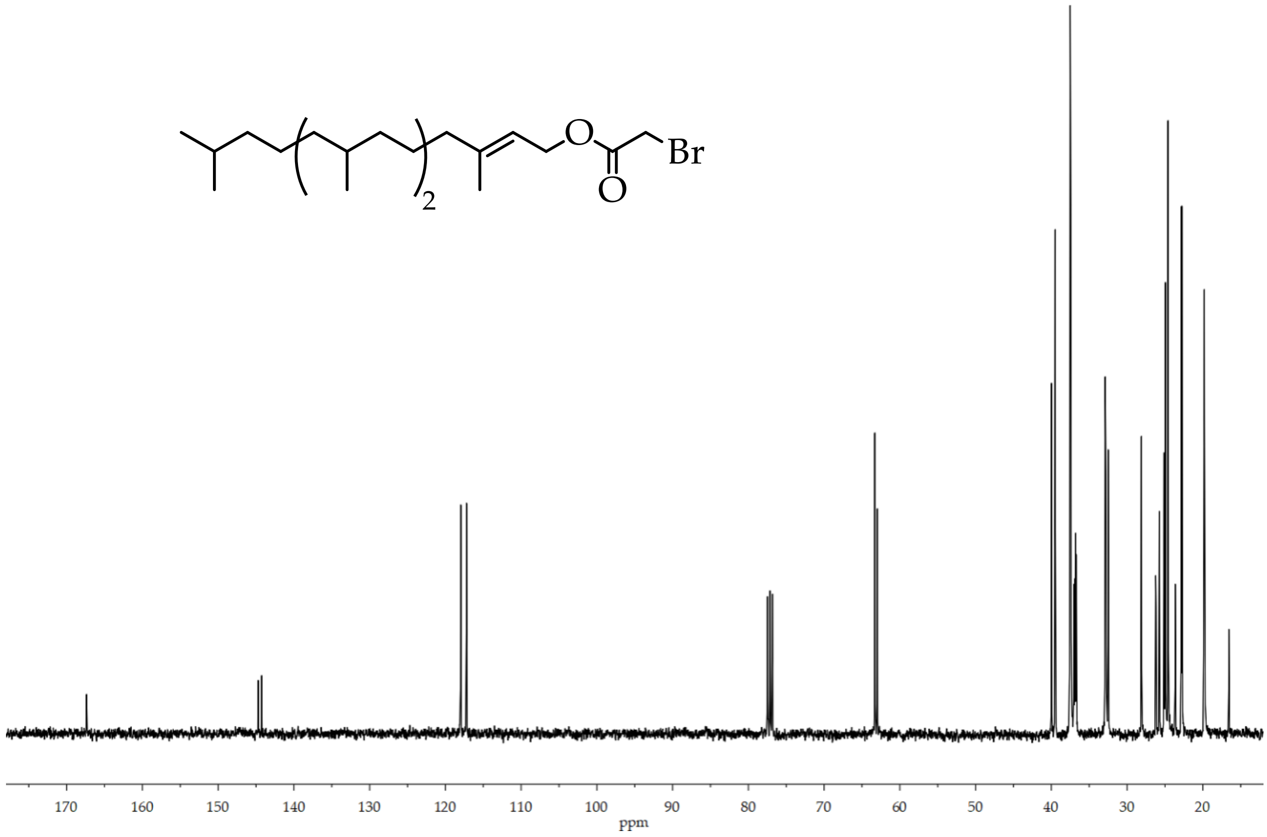

Figure S15. ^{13}C NMR spectrum of the compound **2c**, $CDCl_3$, 298 K, 100 MHz.

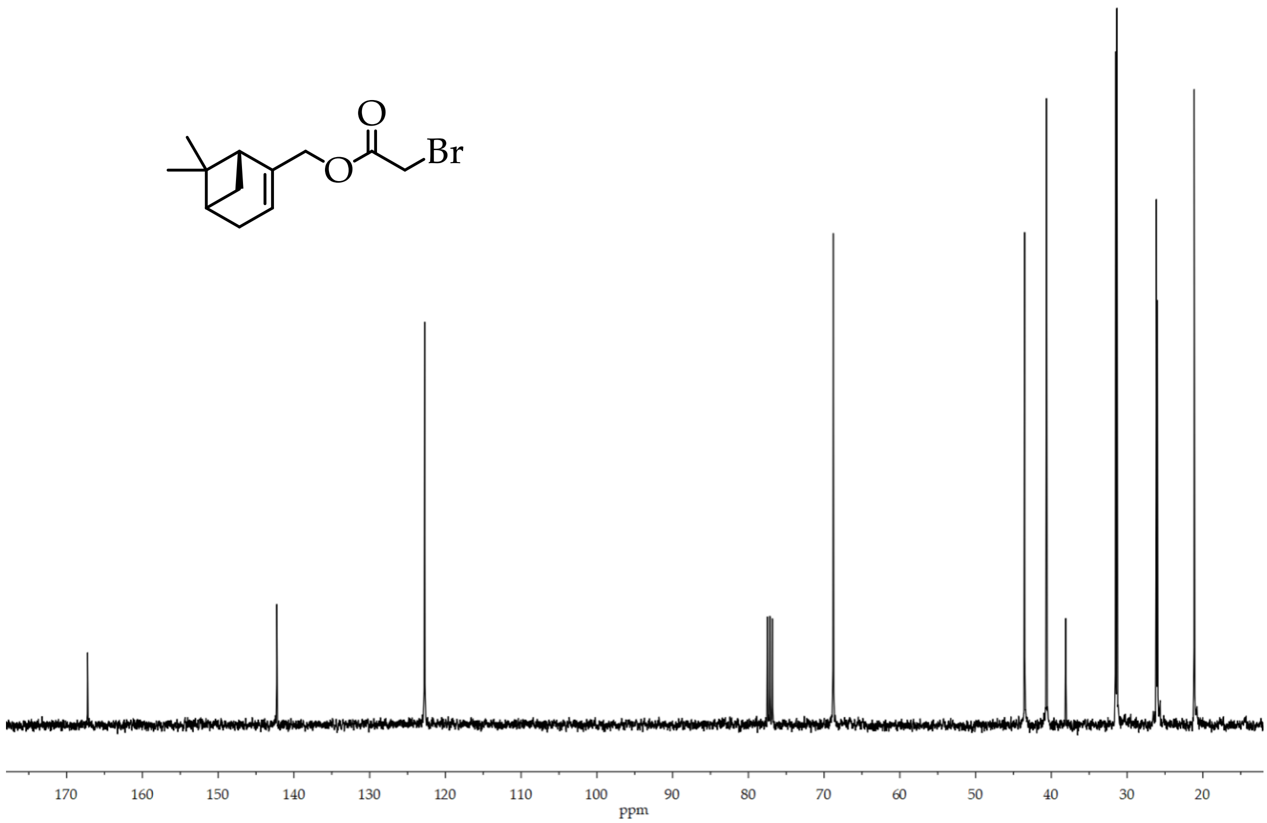

Figure S16. ^{13}C NMR spectrum of the compound **2d**, $CDCl_3$, 298 K, 100 MHz.

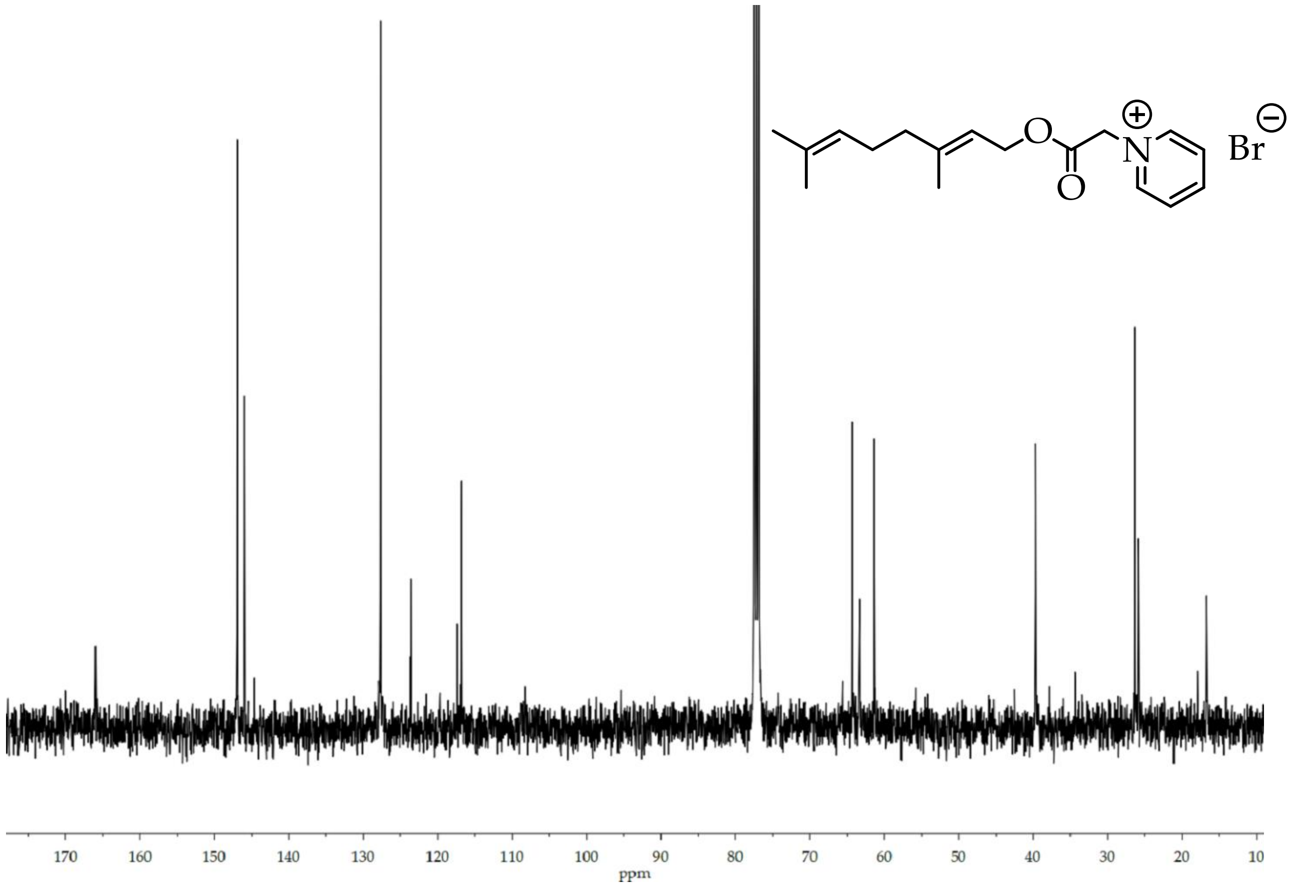

Figure S17. ^{13}C NMR spectrum of the compound **3a**, $CDCl_3$, 298 K, 100 MHz.

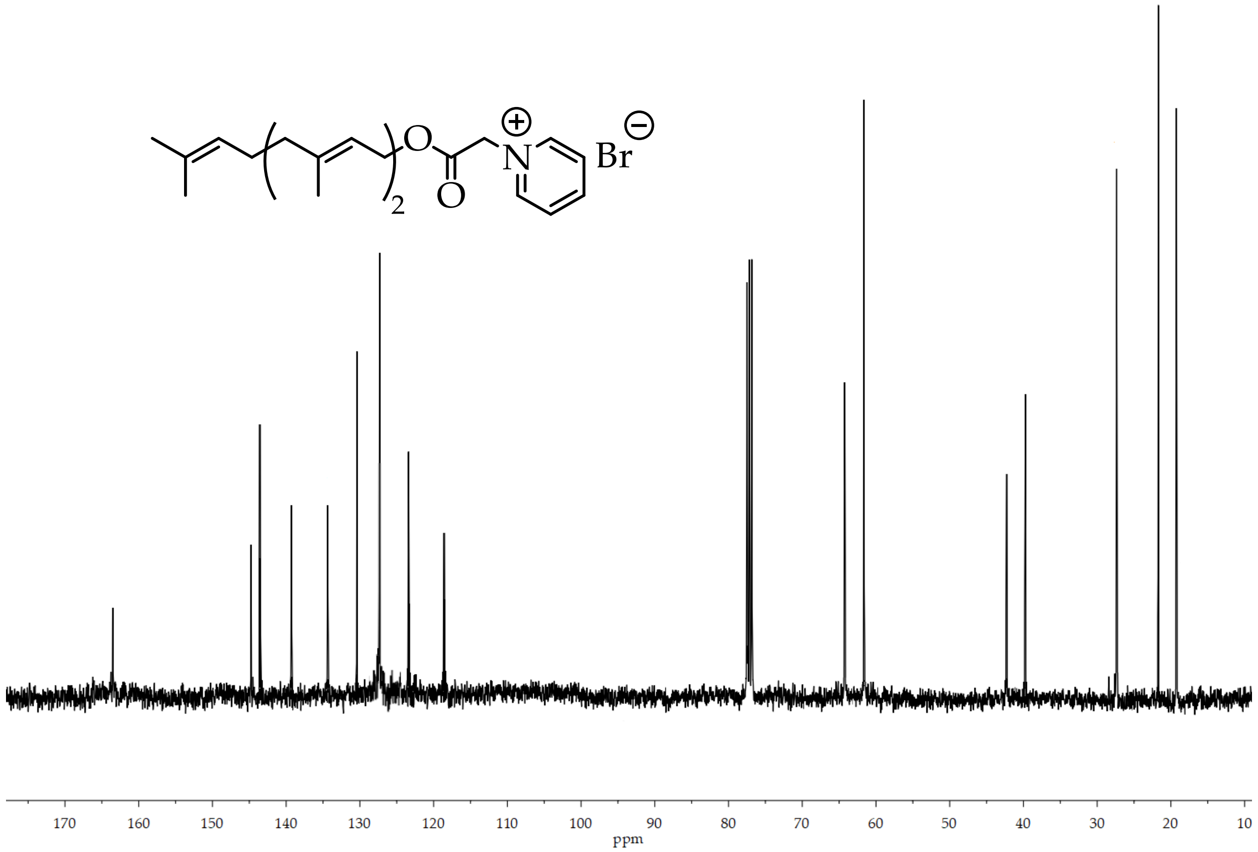

Figure S18. ^{13}C NMR spectrum of the compound **3b**, $CDCl_3$, 298 K, 100 MHz.

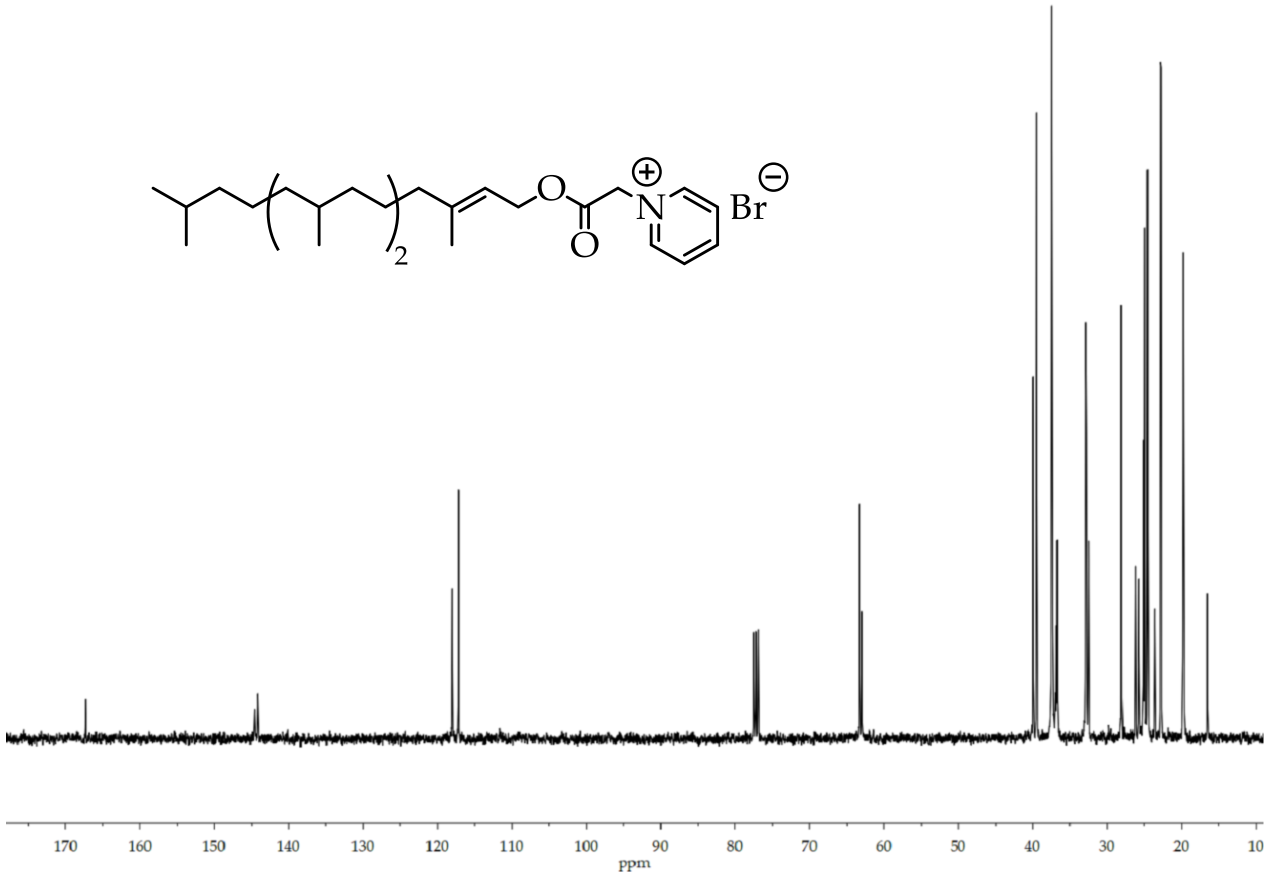

Figure S19. ^{13}C NMR spectrum of the compound **3c**, $CDCl_3$, 298 K, 100 MHz.

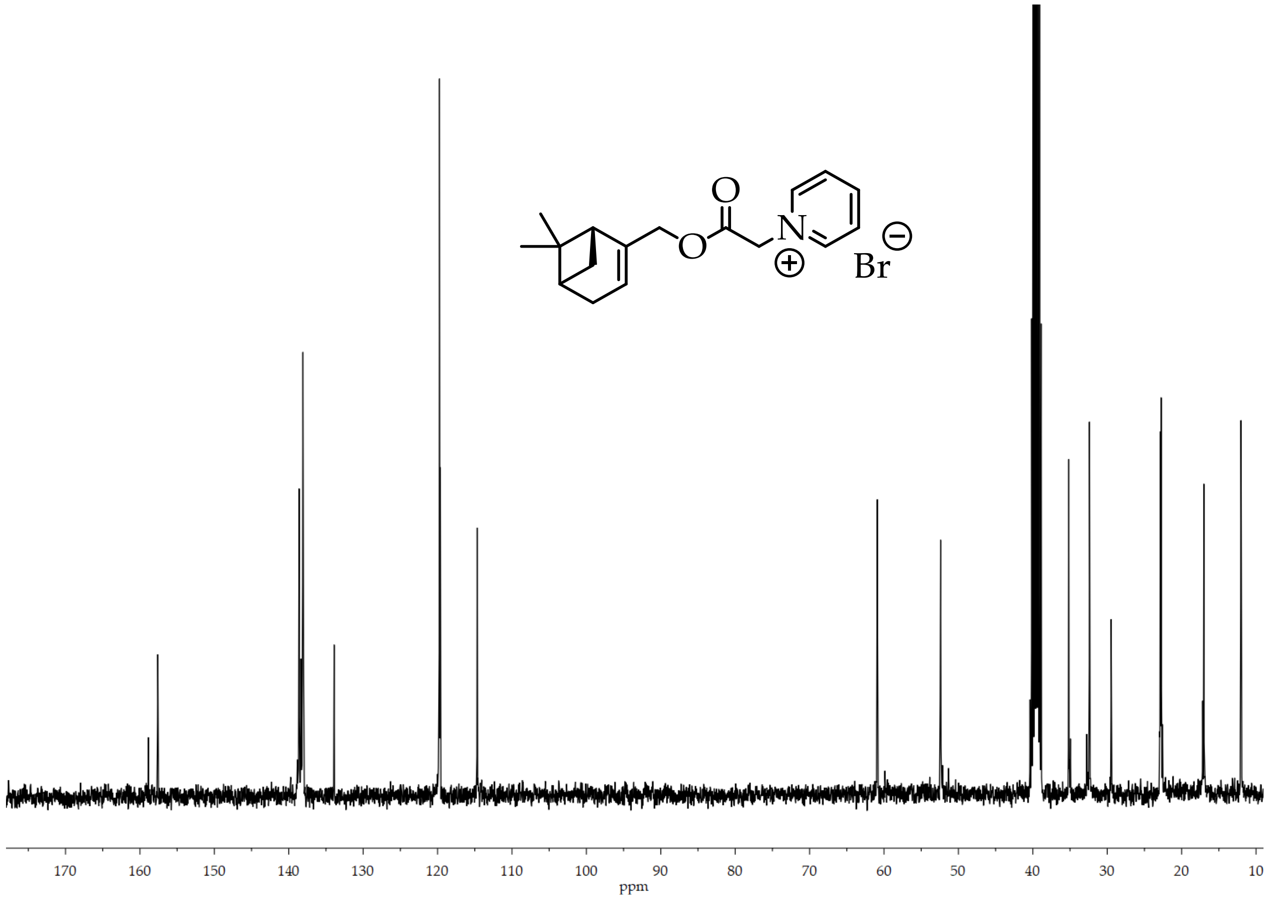

Figure S20. ^{13}C NMR spectrum of the compound **3d**, DMSO*d6*, 298 K, 100 MHz.

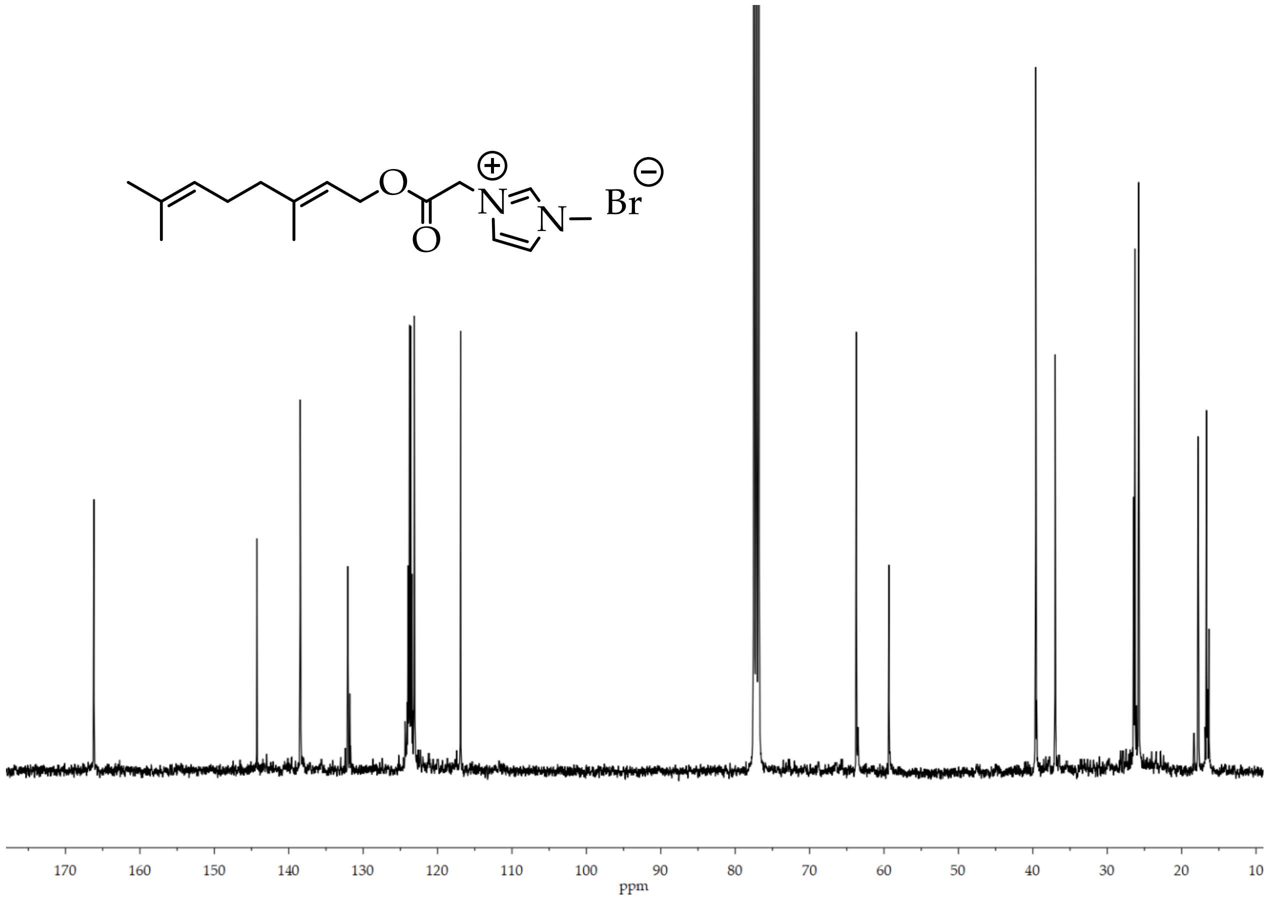

Figure S21. ^{13}C NMR spectrum of the compound **4a**, $CDCl_3$, 298 K, 100 MHz.

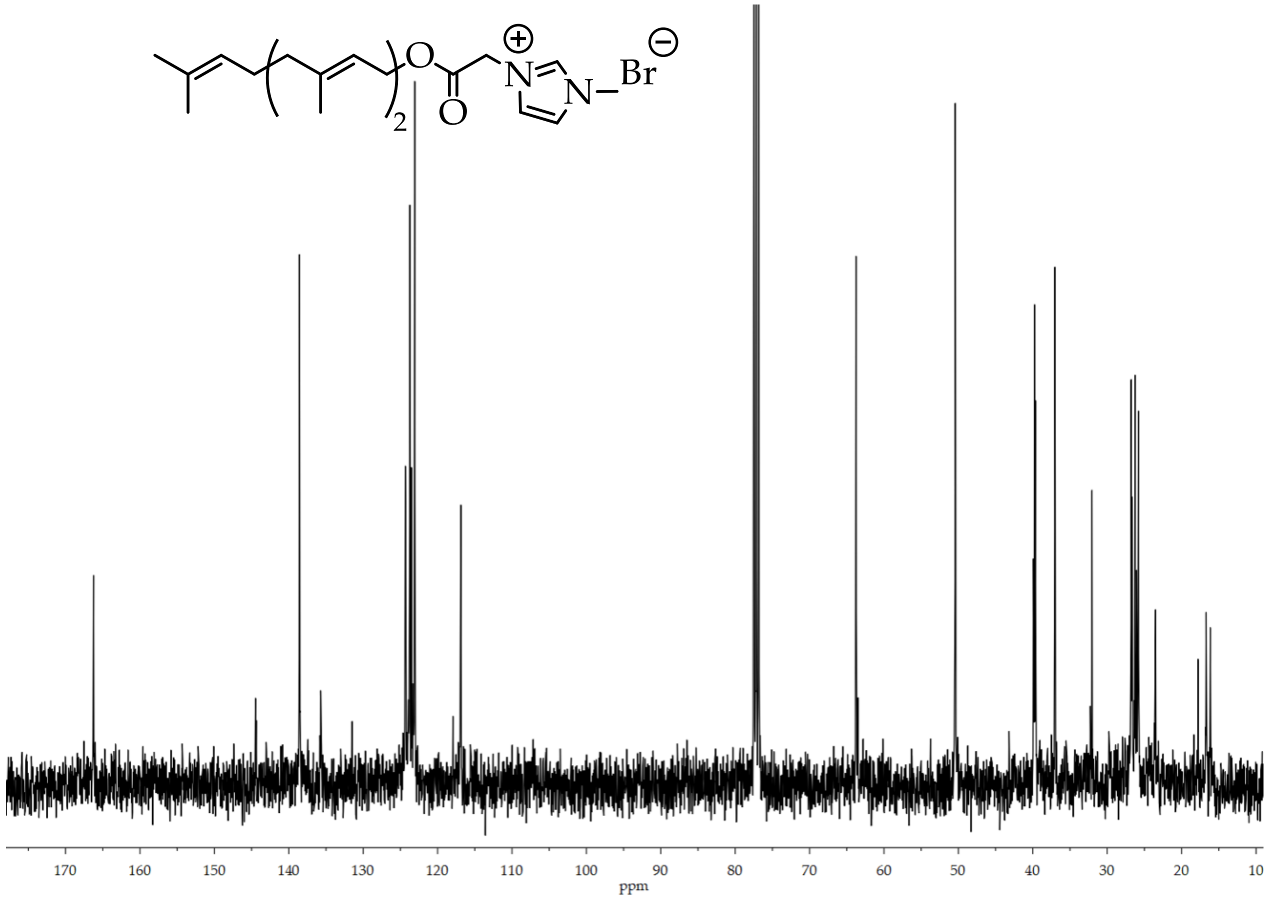

Figure S22. ^{13}C NMR spectrum of the compound **4b**, $CDCl_3$, 298 K, 100 MHz.

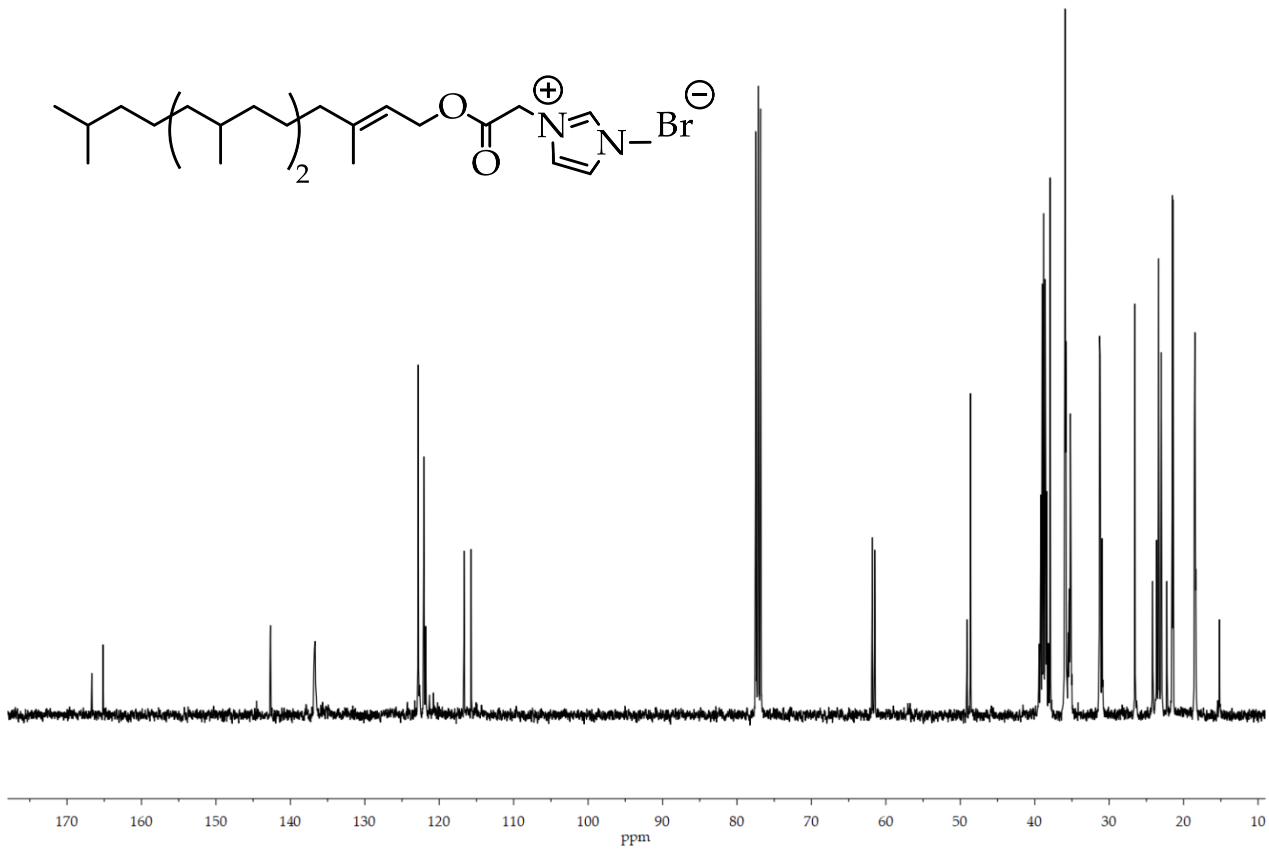

Figure S23. ^{13}C NMR spectrum of the compound **4c**, $CDCl_3$, 298 K, 100 MHz.

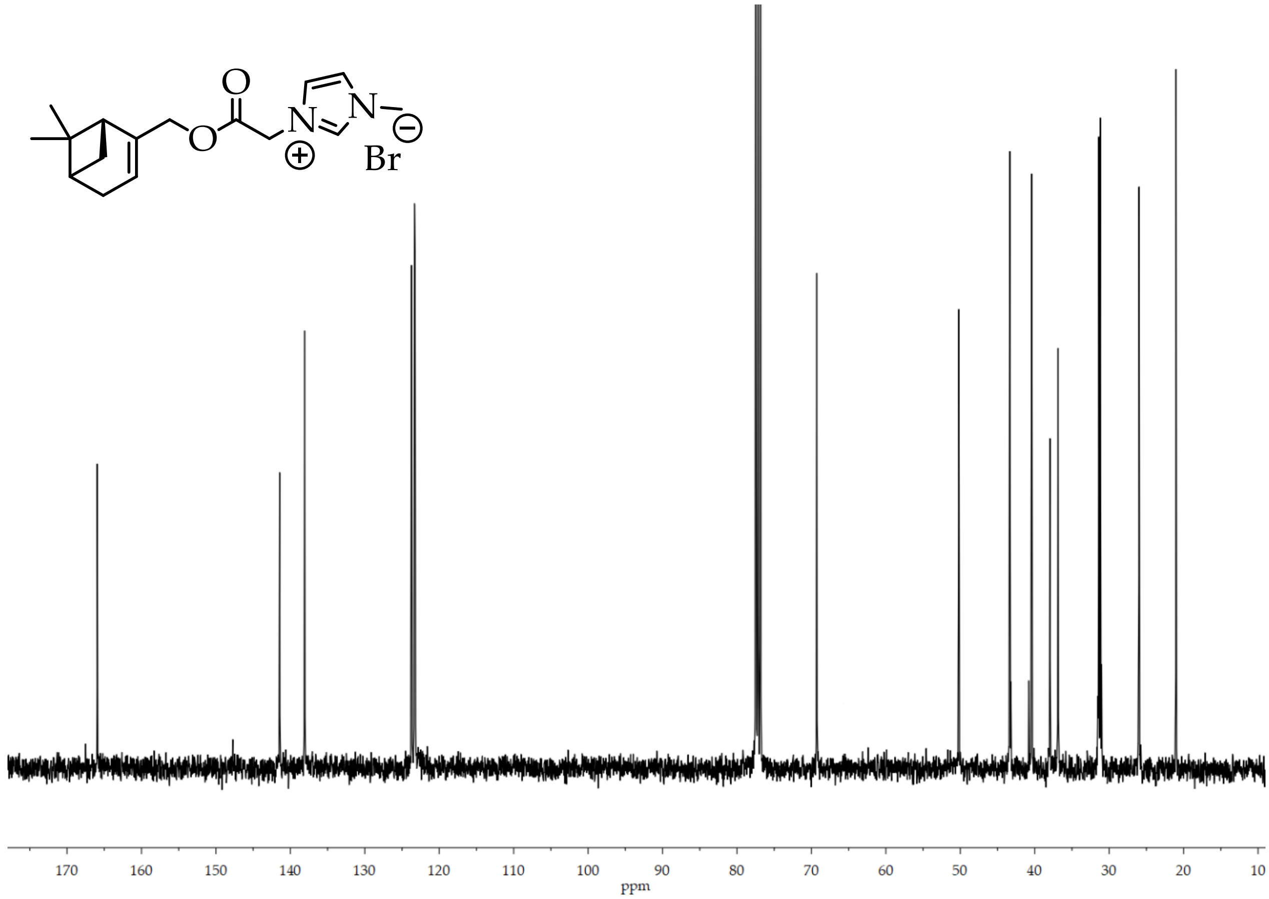

Figure S24. ^{13}C NMR spectrum of the compound **4d**, $CDCl_3$, 298 K, 100 MHz.

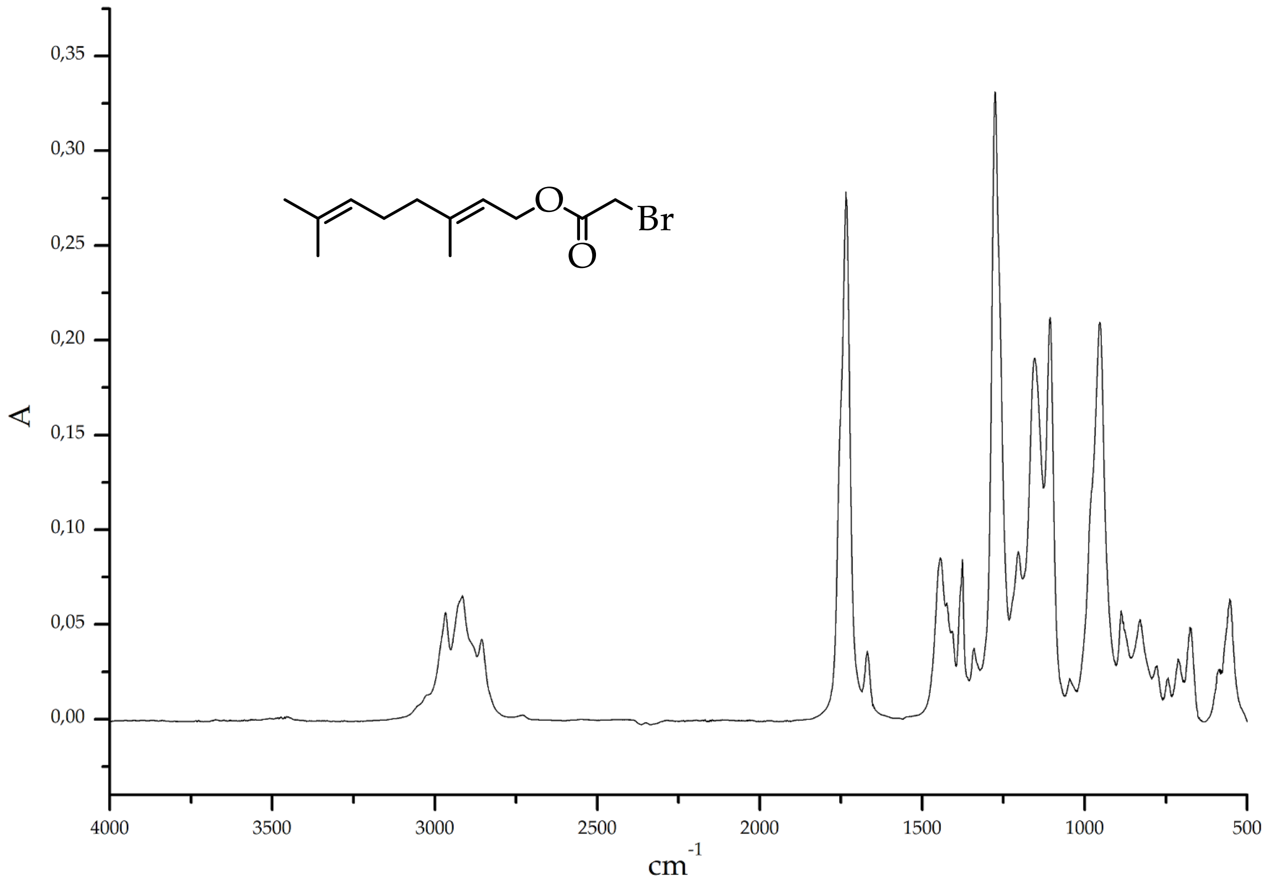

Figure S25. FT-IR spectrum of the compound **2a**.

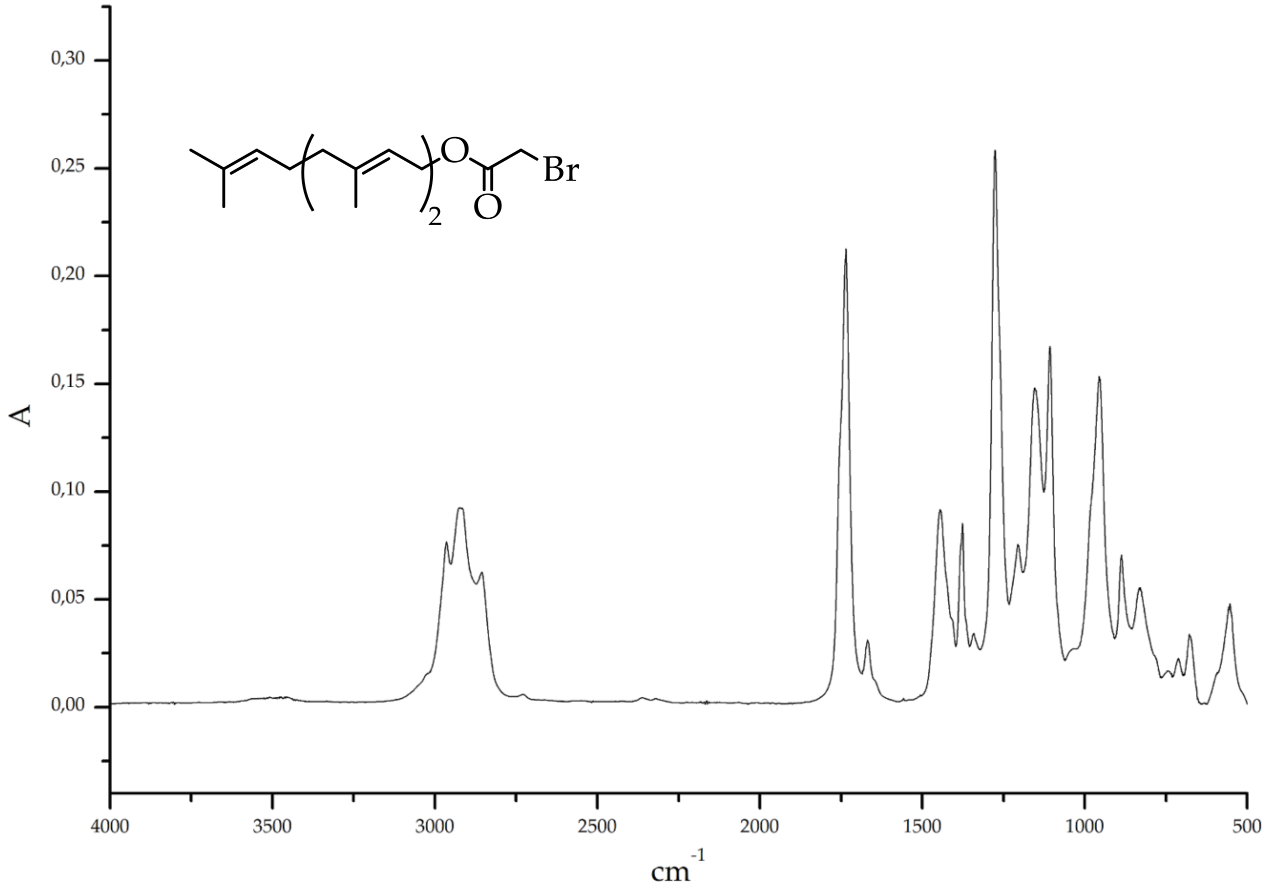

Figure S26. FT-IR spectrum of the compound **2b**.

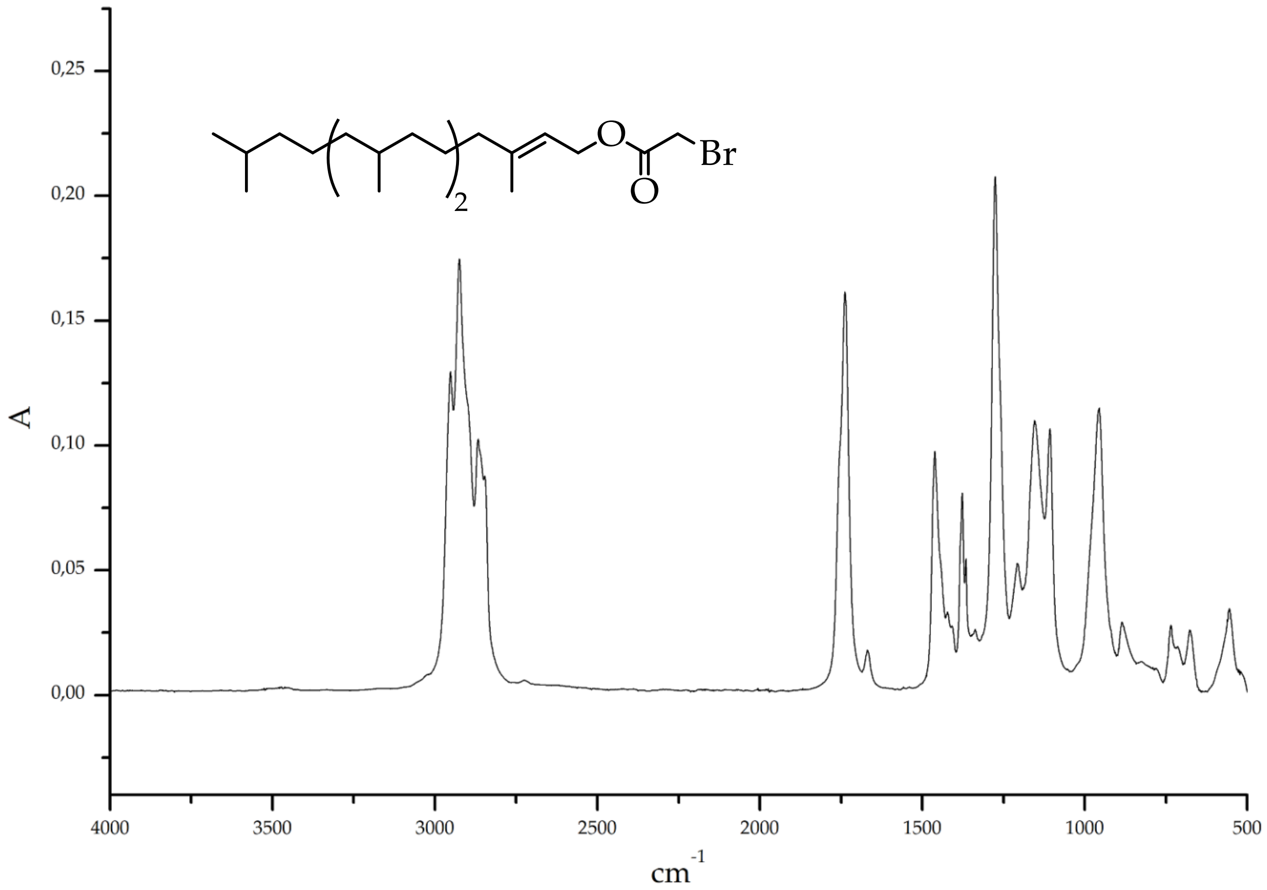

Figure S27. FT-IR spectrum of the compound **2c**.

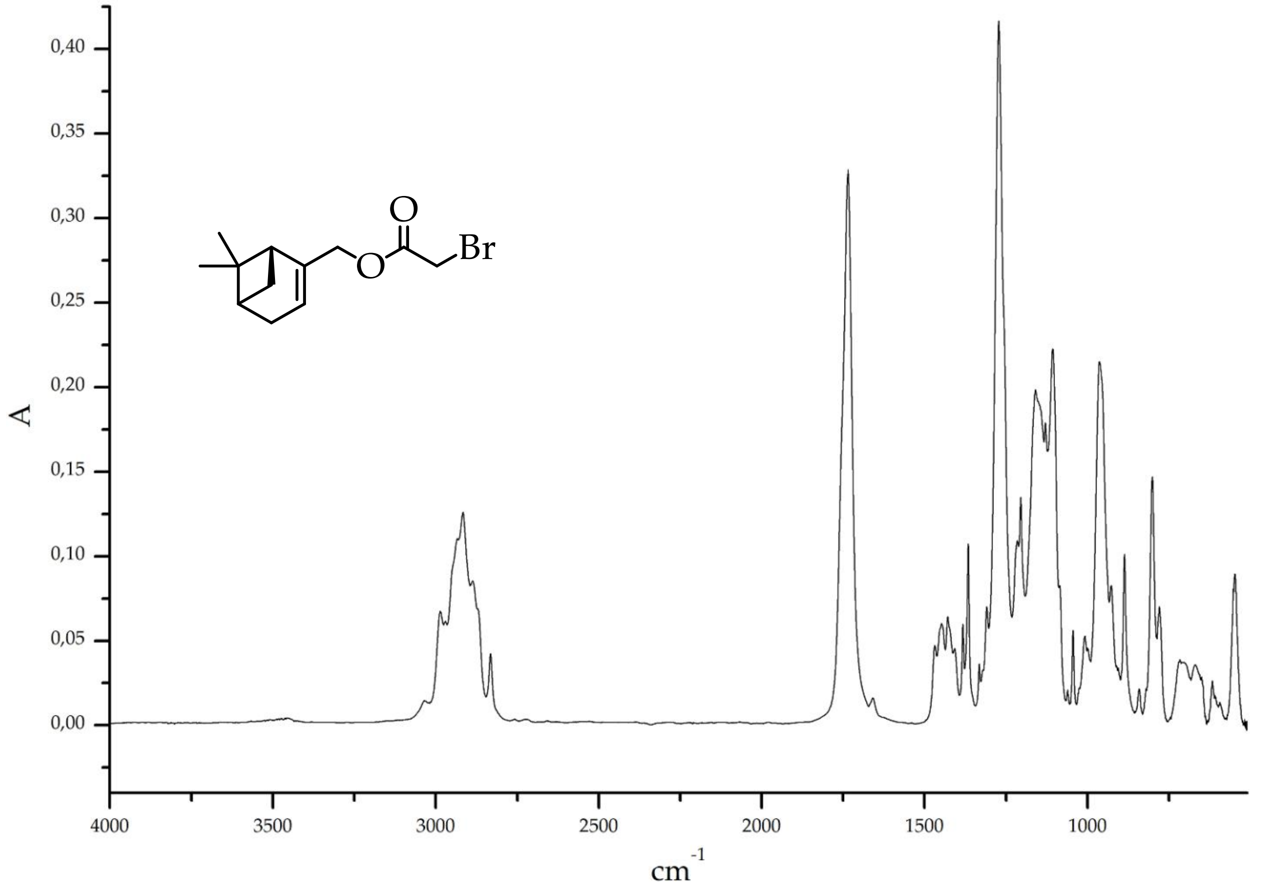

Figure S28. FT-IR spectrum of the compound **2d**.

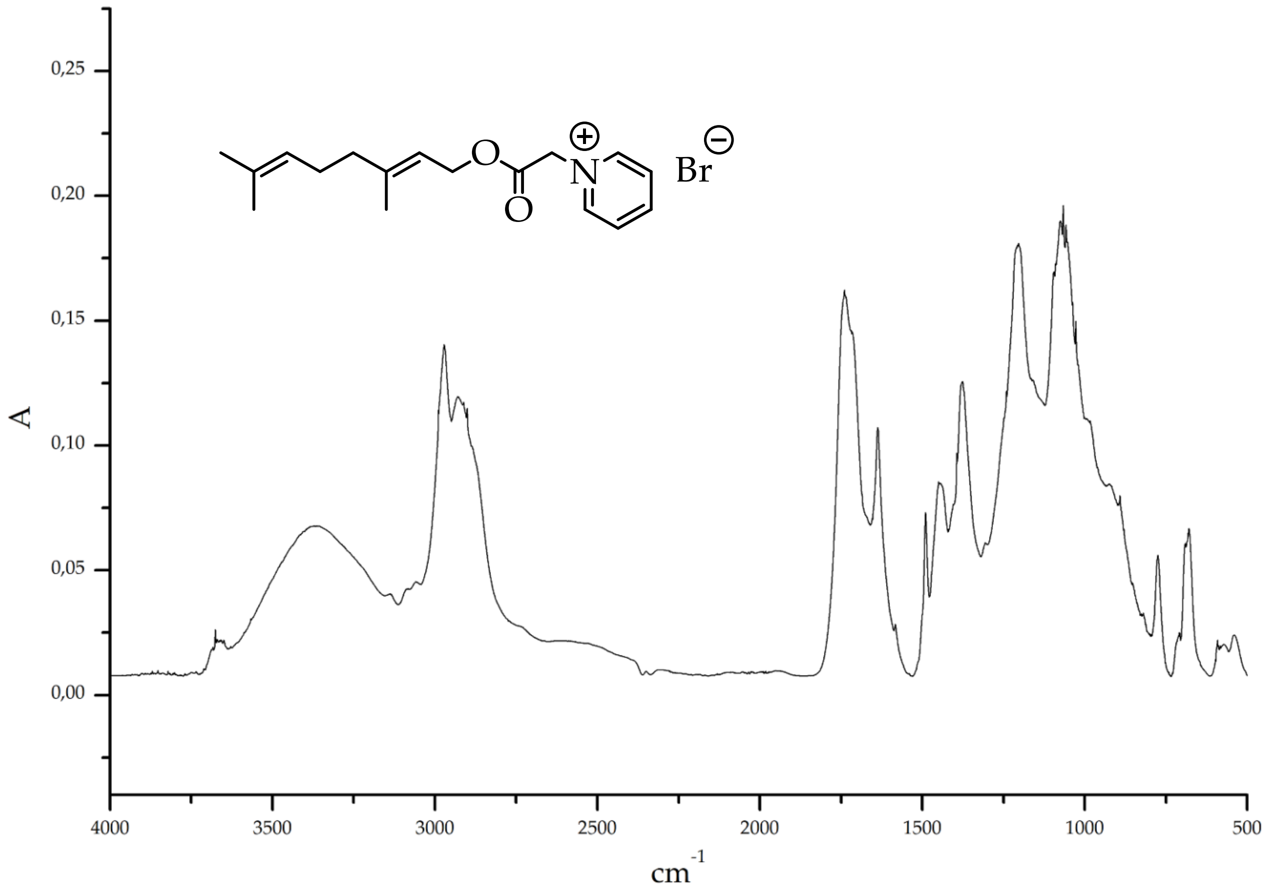

Figure S29. FT-IR spectrum of the compound **3a**.

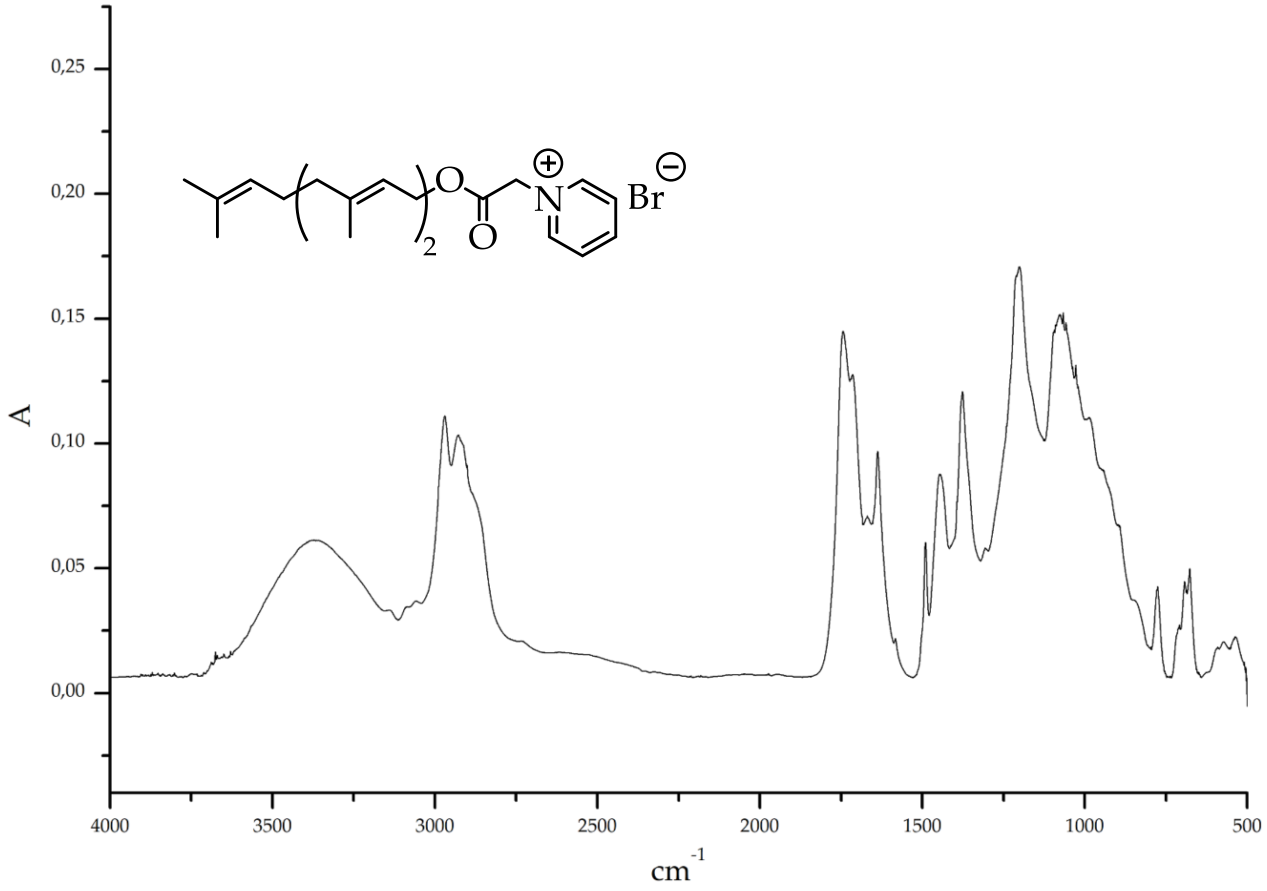

Figure S30. FT-IR spectrum of the compound **3b**.

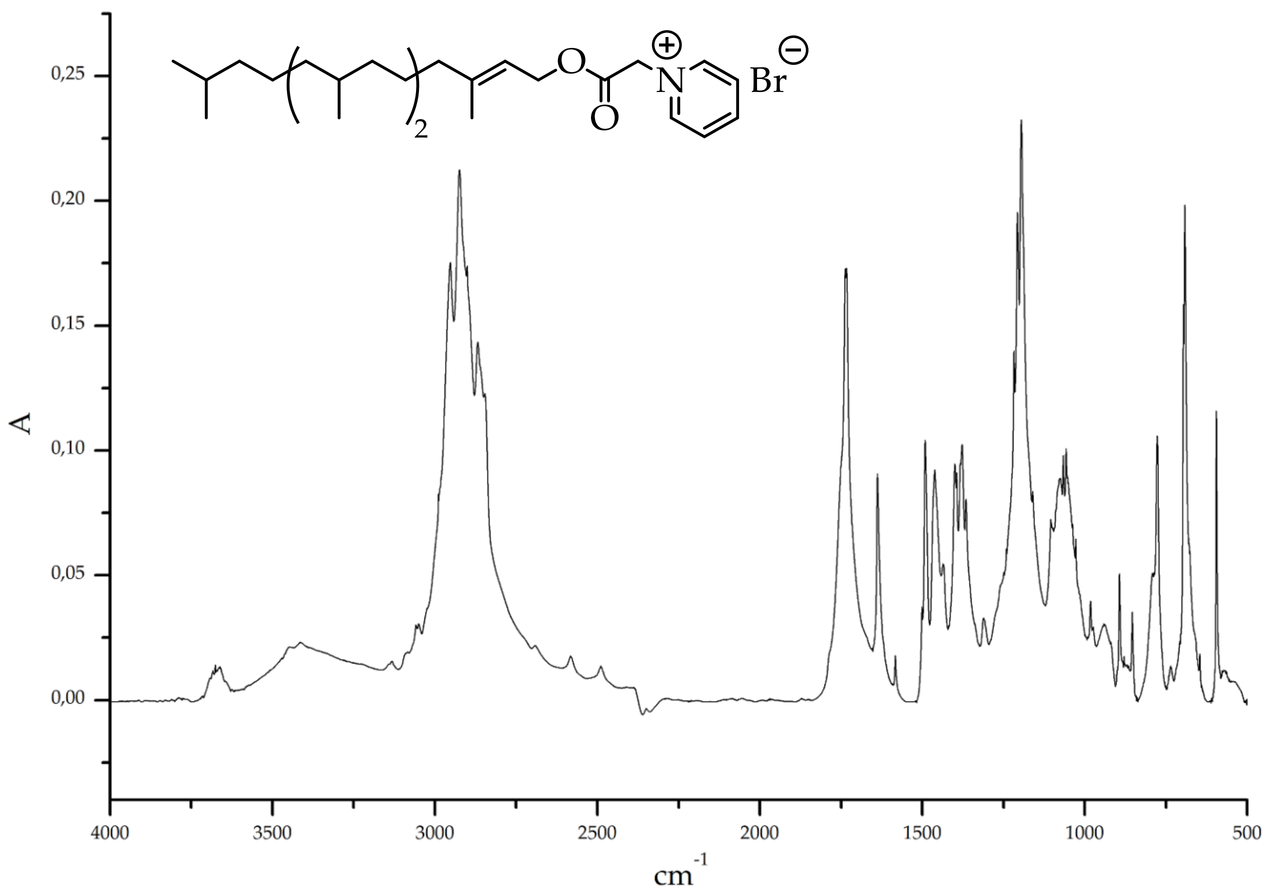

Figure S31. FT-IR spectrum of the compound **3c**.

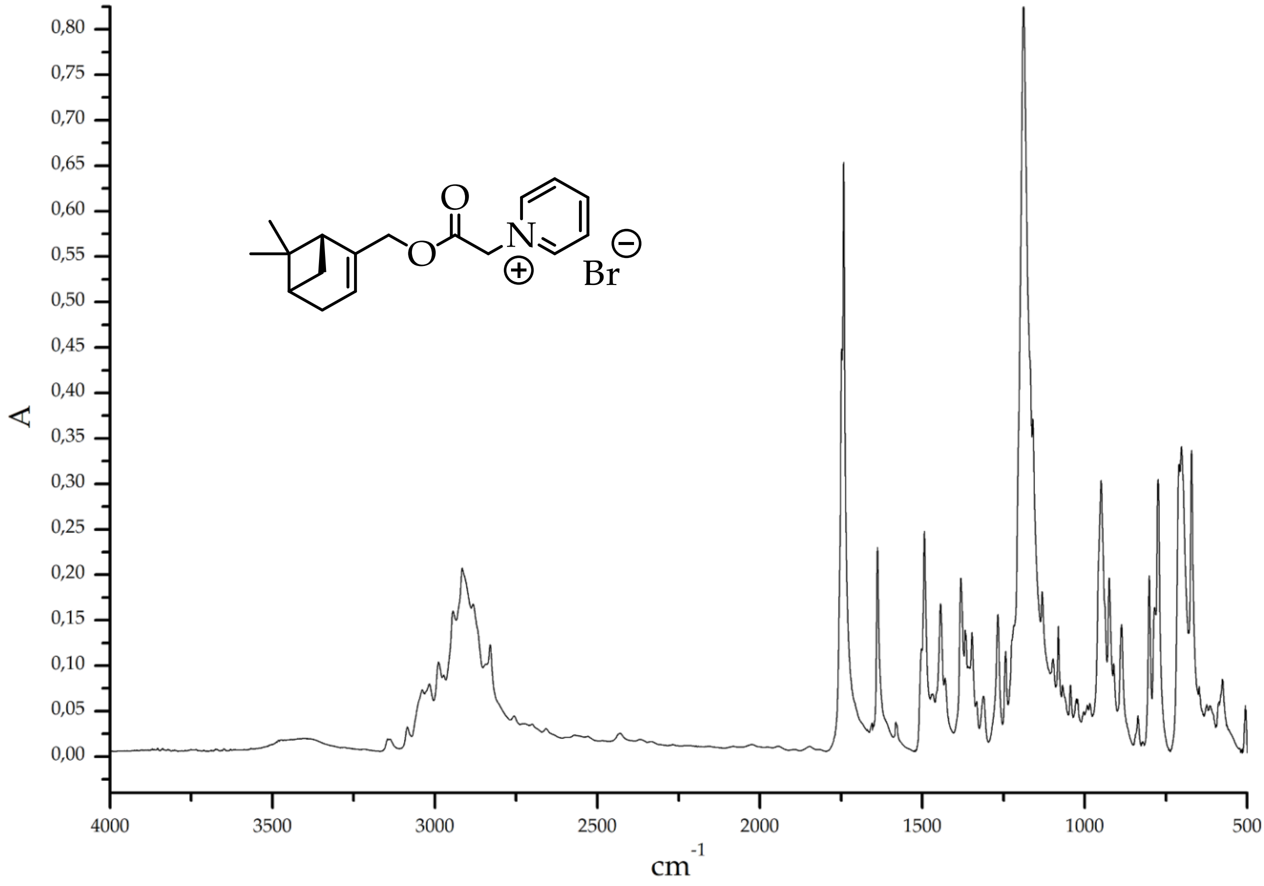

Figure S32. FT-IR spectrum of the compound **3d**.

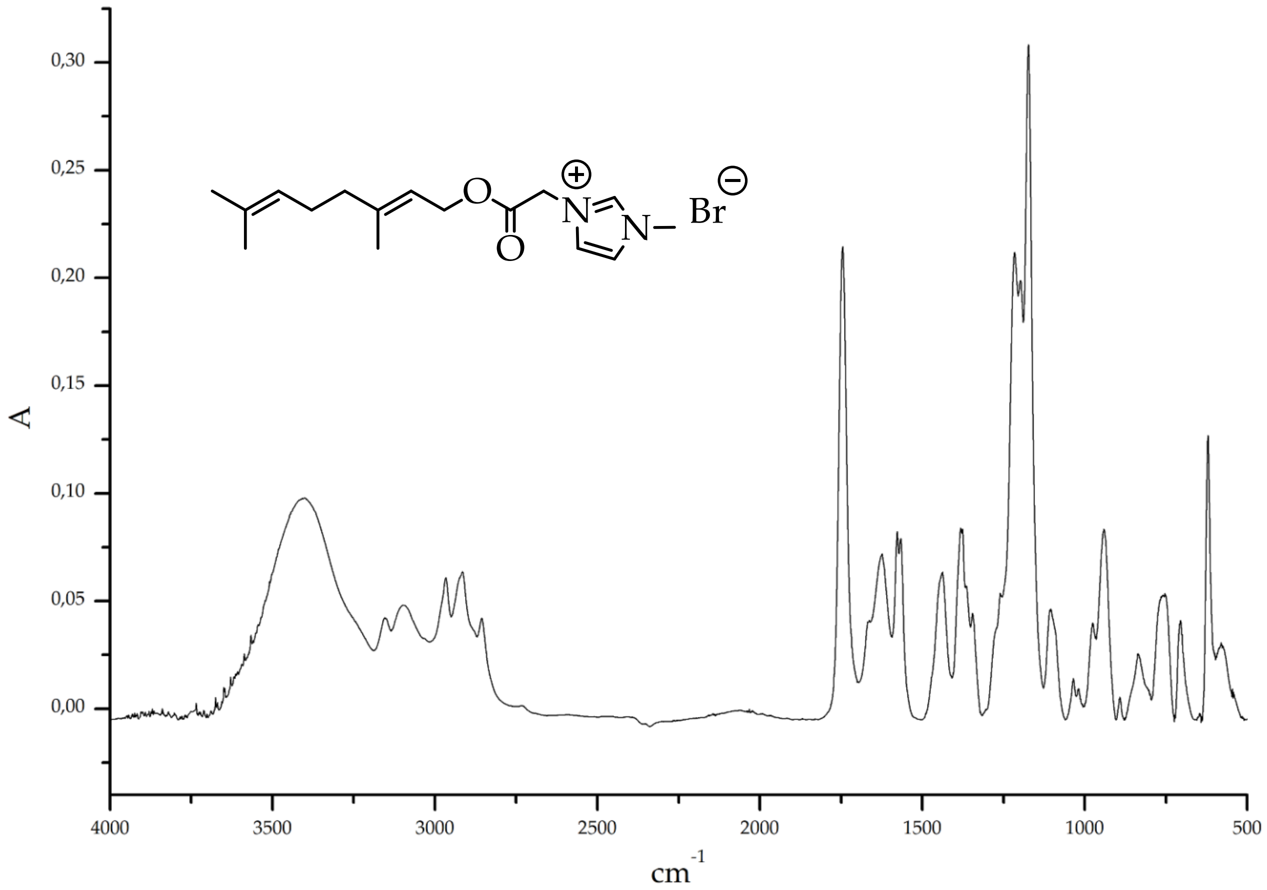

Figure S33. FT-IR spectrum of the compound **4a**.

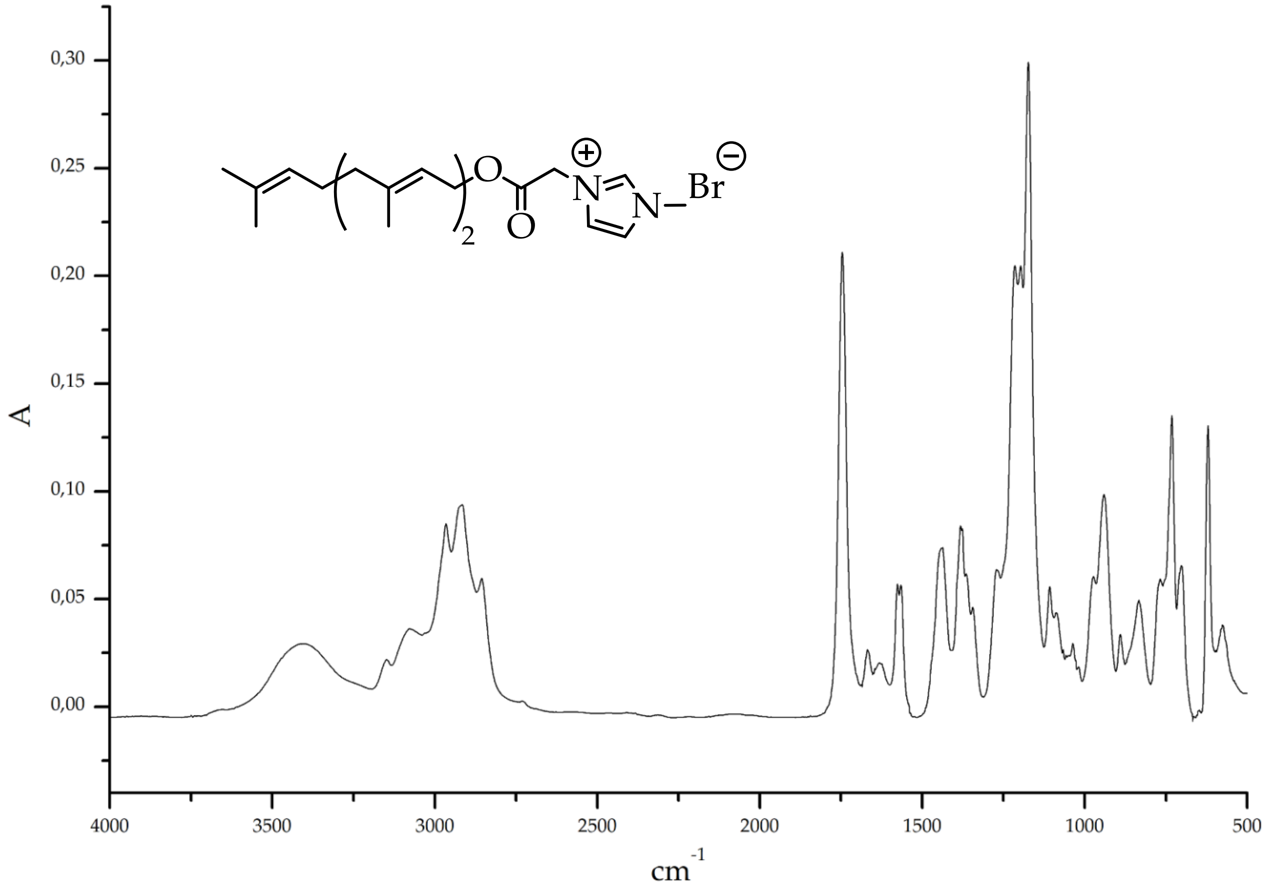

Figure S34. FT-IR spectrum of the compound **4b**.

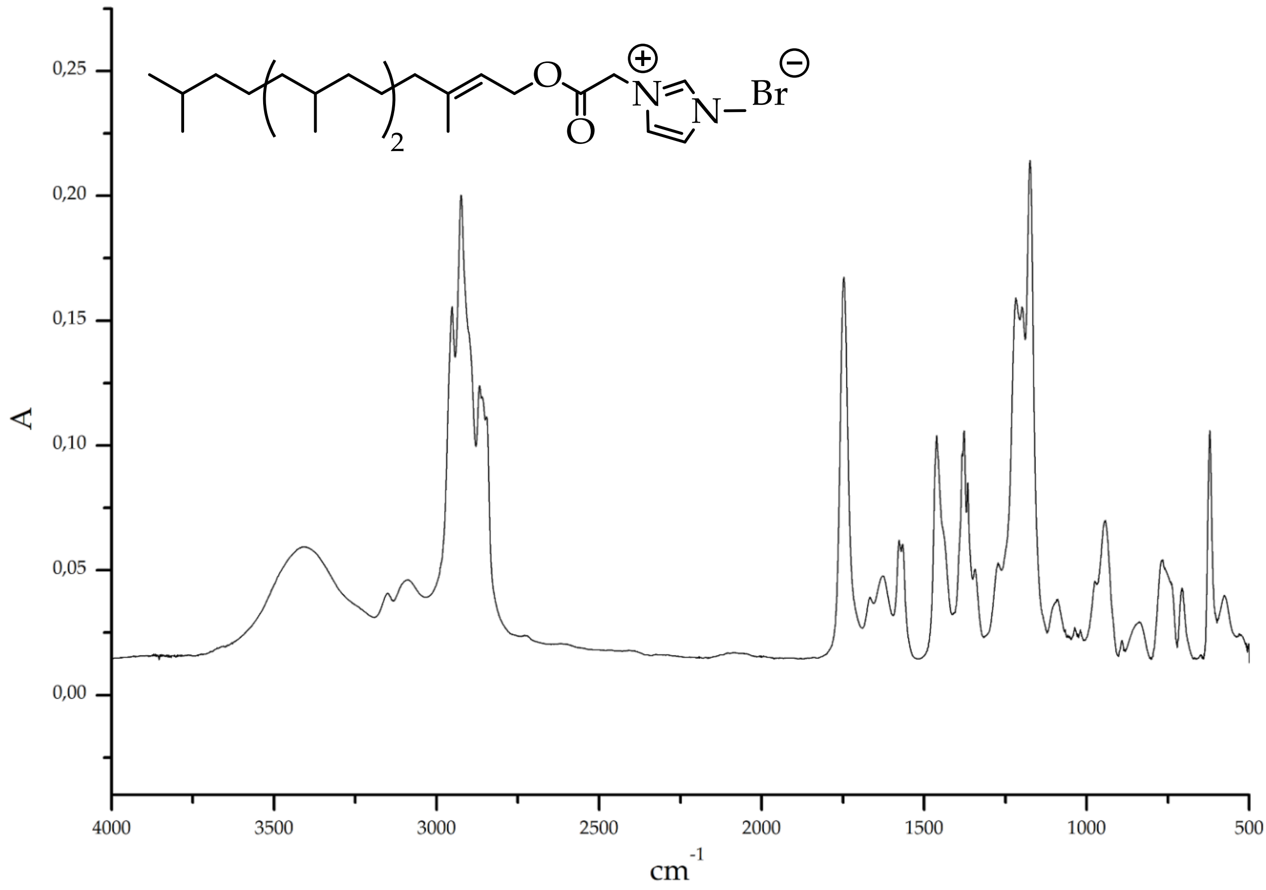

Figure S35. FT-IR spectrum of the compound **4c**.

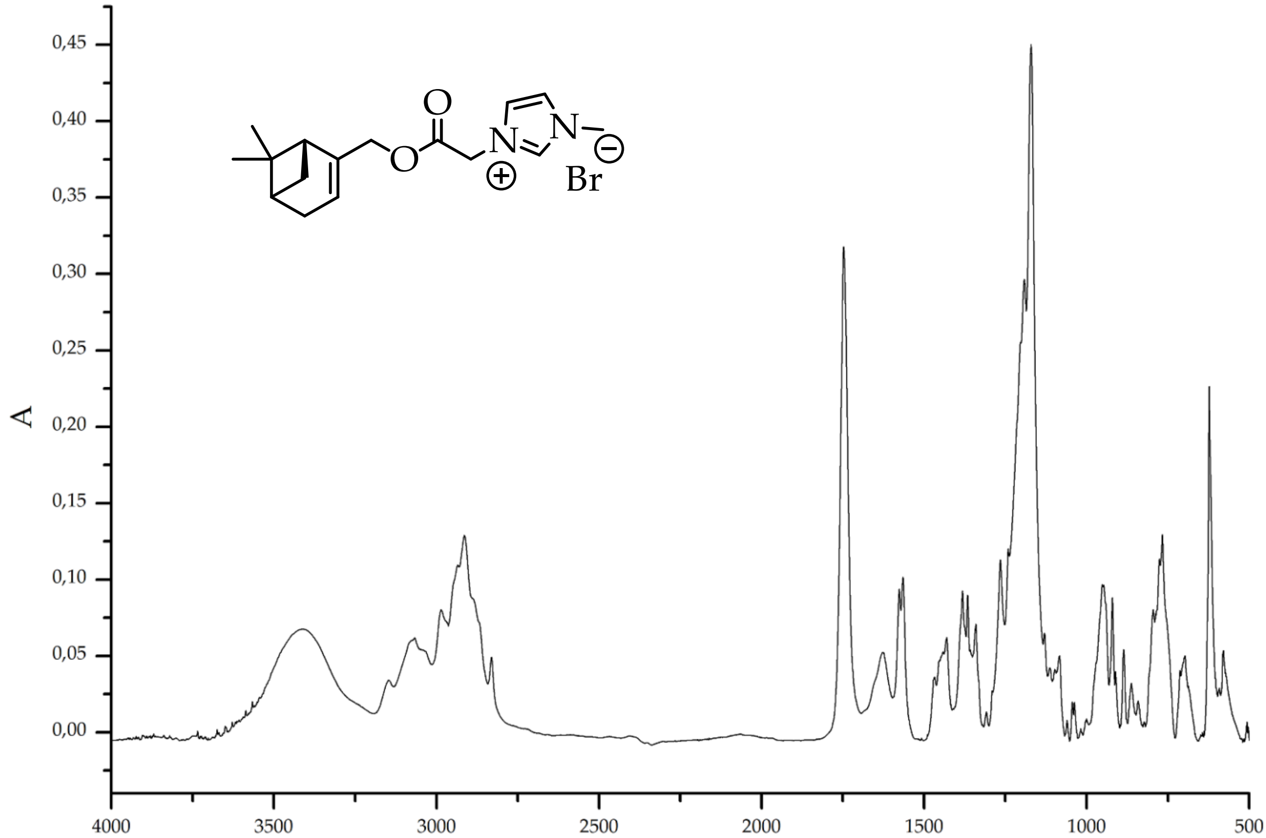

Figure S36. FT-IR spectrum of the compound **4d**.

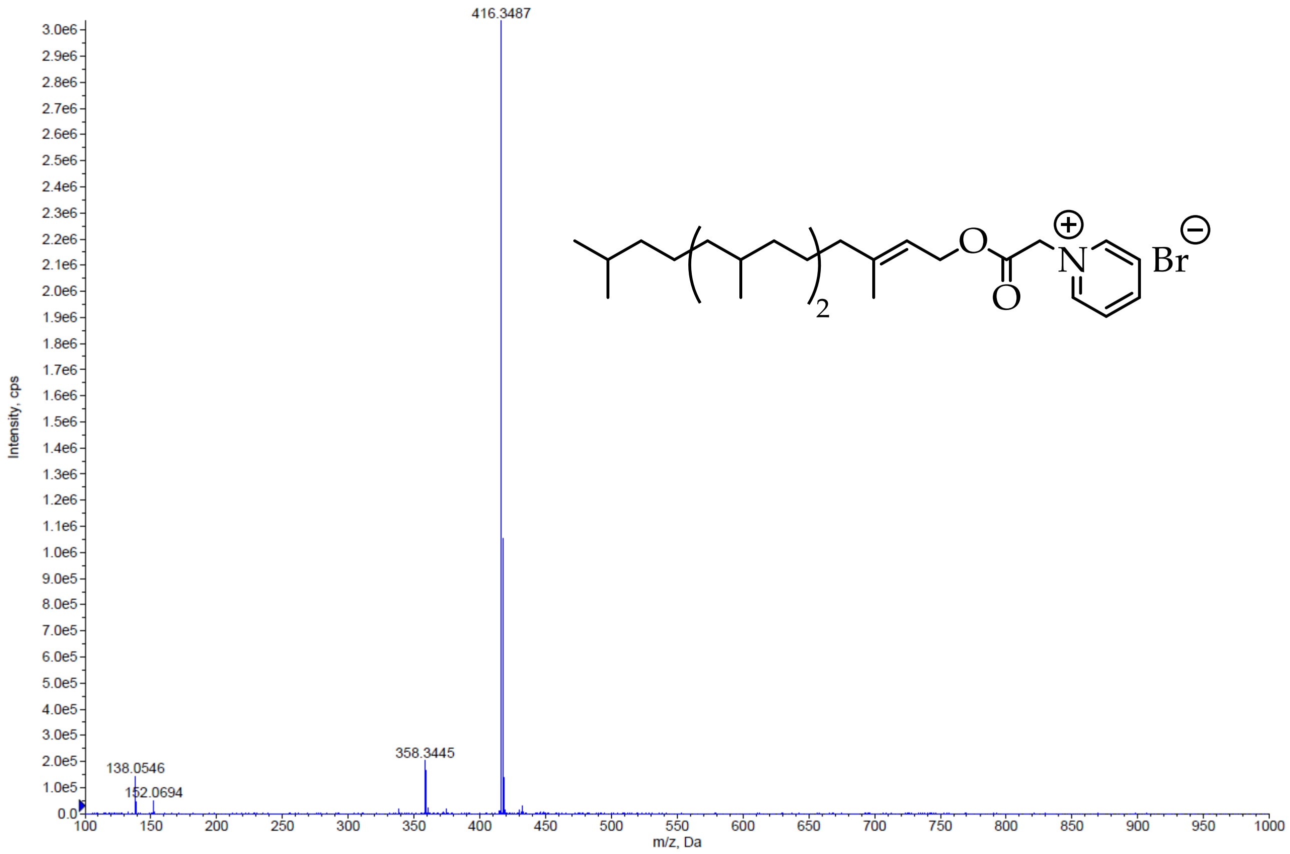

Figure S37. HRMS spectrum of the compound **3c**.

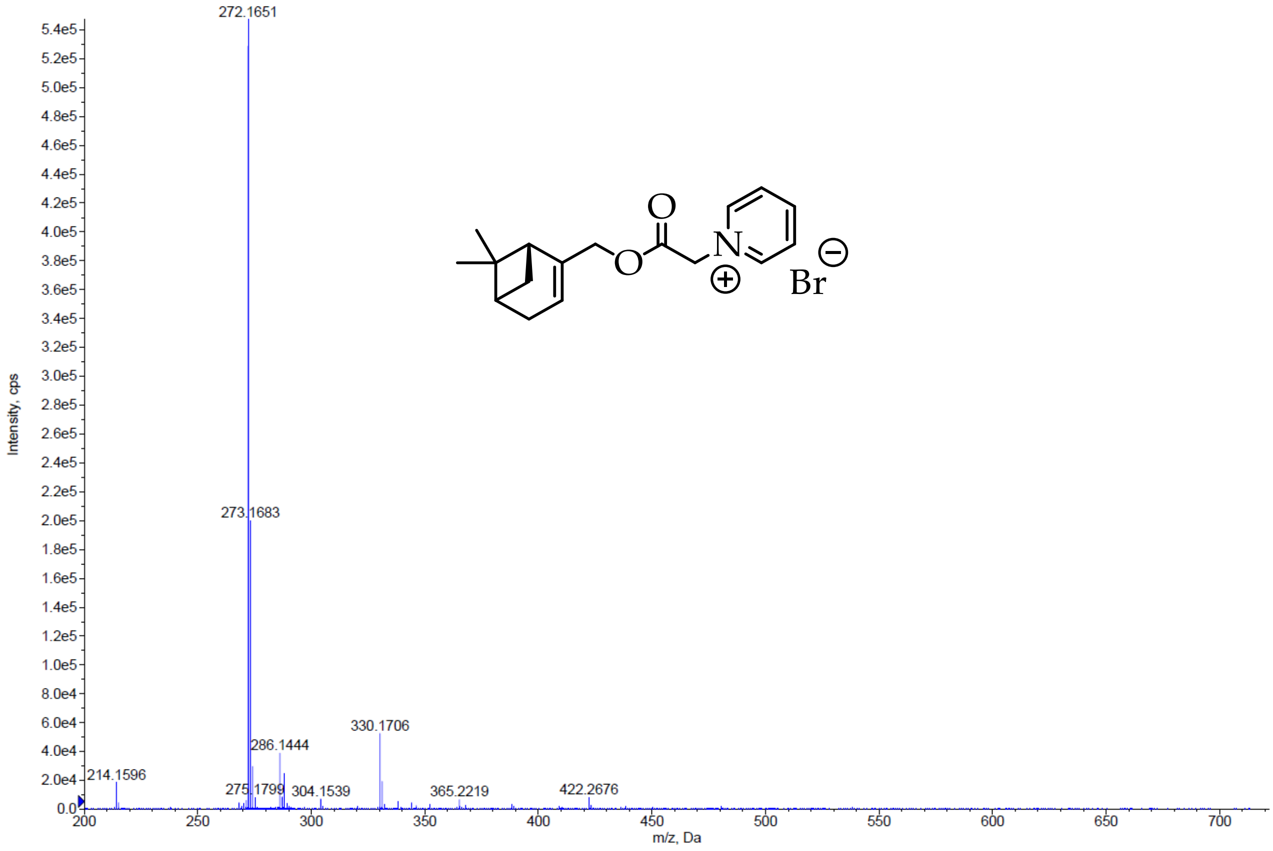

Figure S38. HRMS spectrum of the compound **3d**.

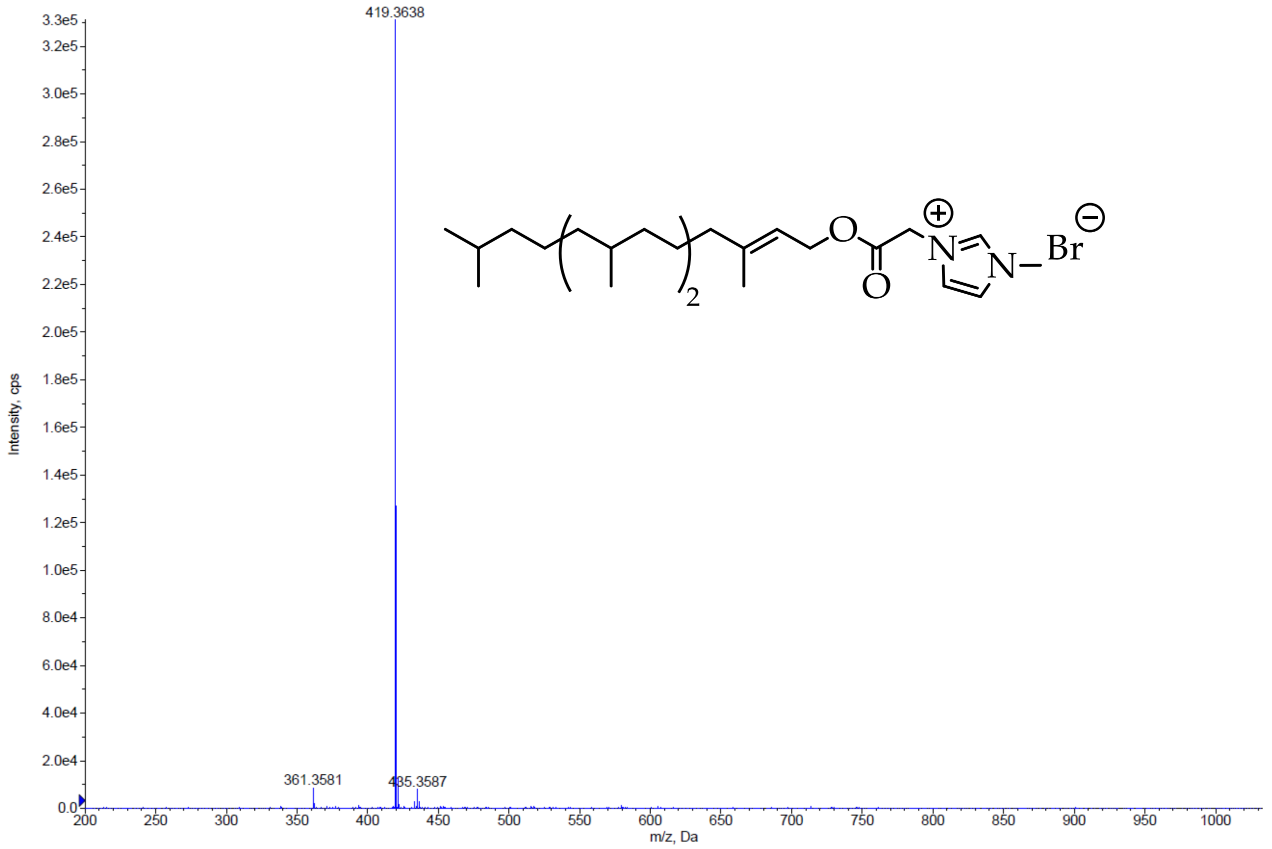

Figure S39. HRMS spectrum of the compound **4c**.

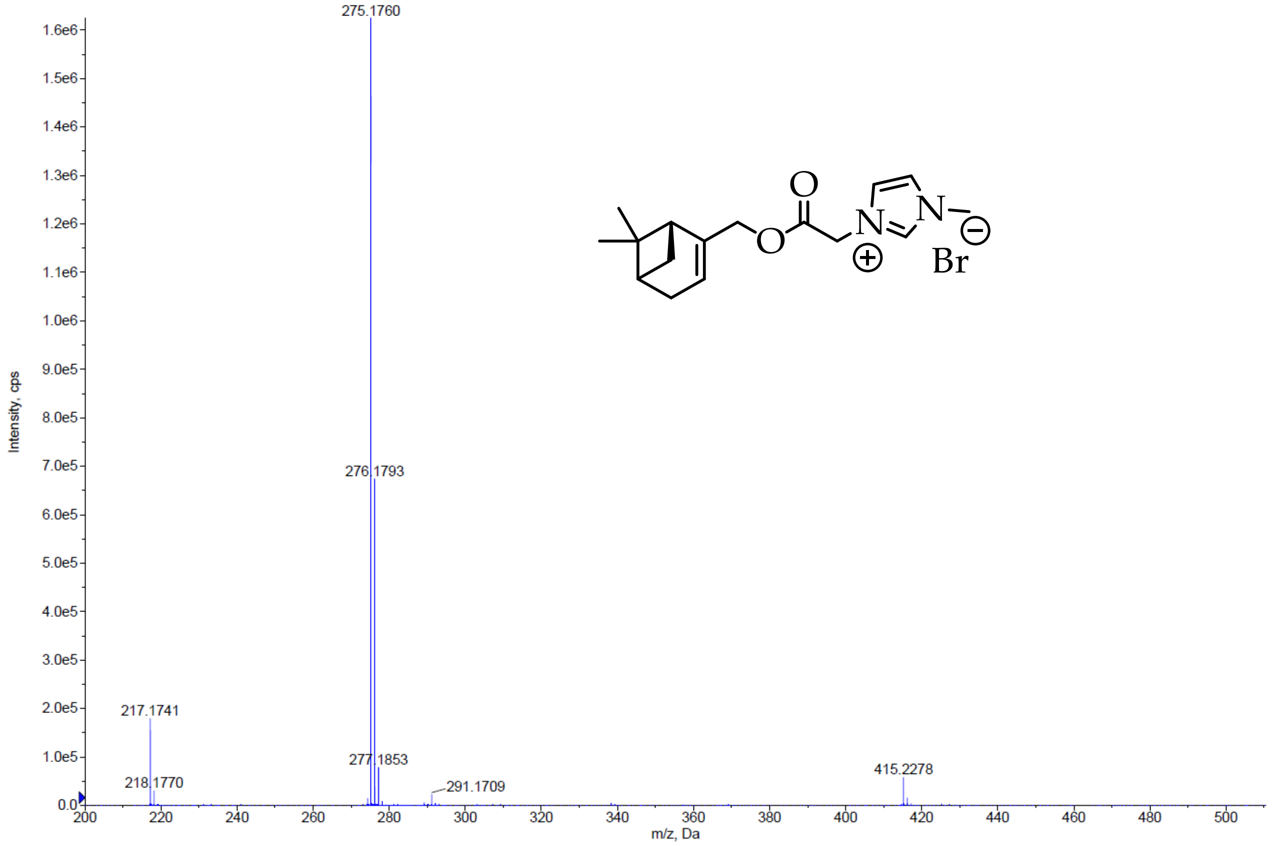

Figure S40. HRMS spectrum of the compound **4d**.

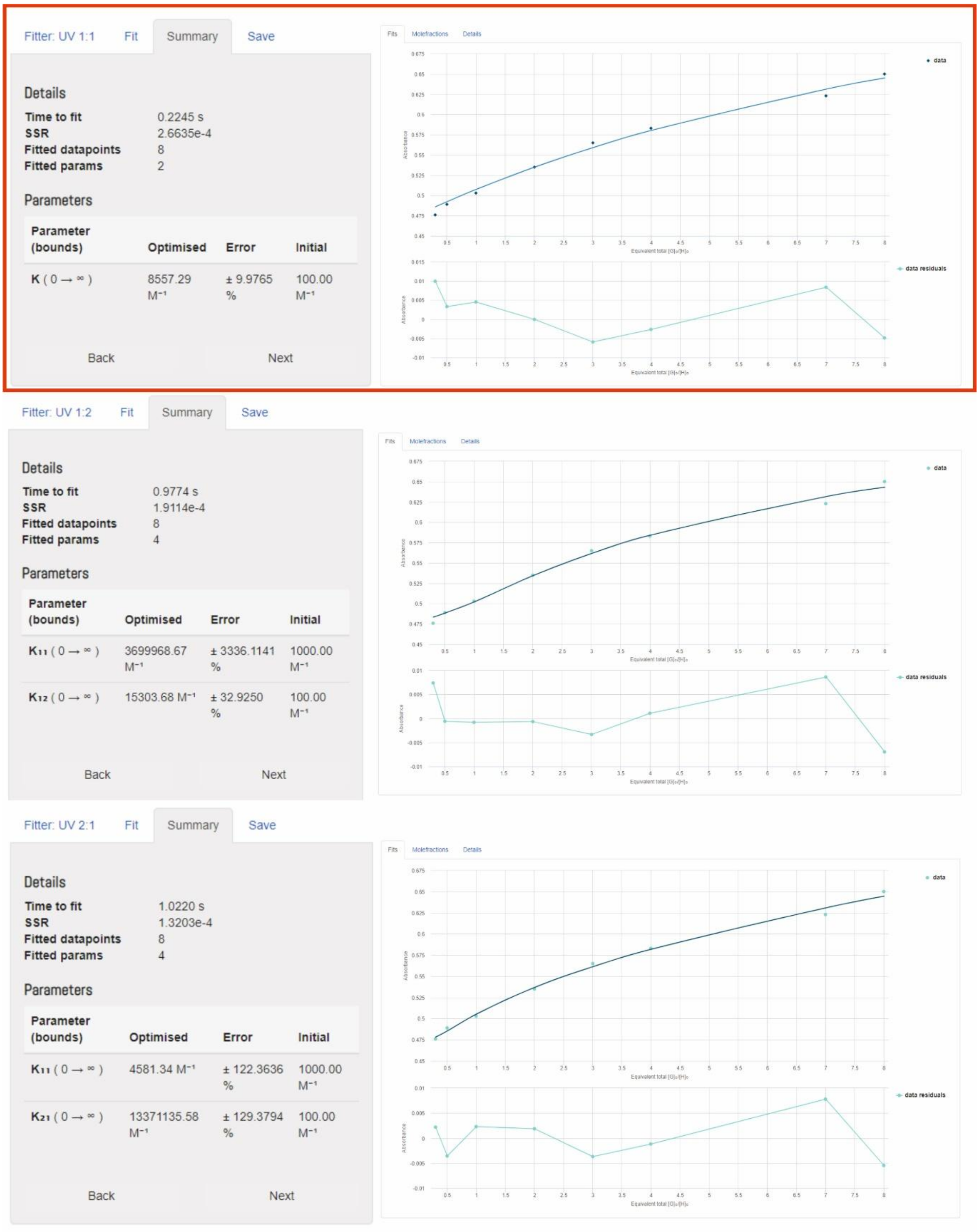

Figure S41. Bindfit (Fit data to 1:1, 1:2 and 2:1 Host-Guest equilibria) Screenshots taken from the summary window of the website supramolecular.org. This screenshots shows the raw data for UV-vis titration of **5** with **3a**.

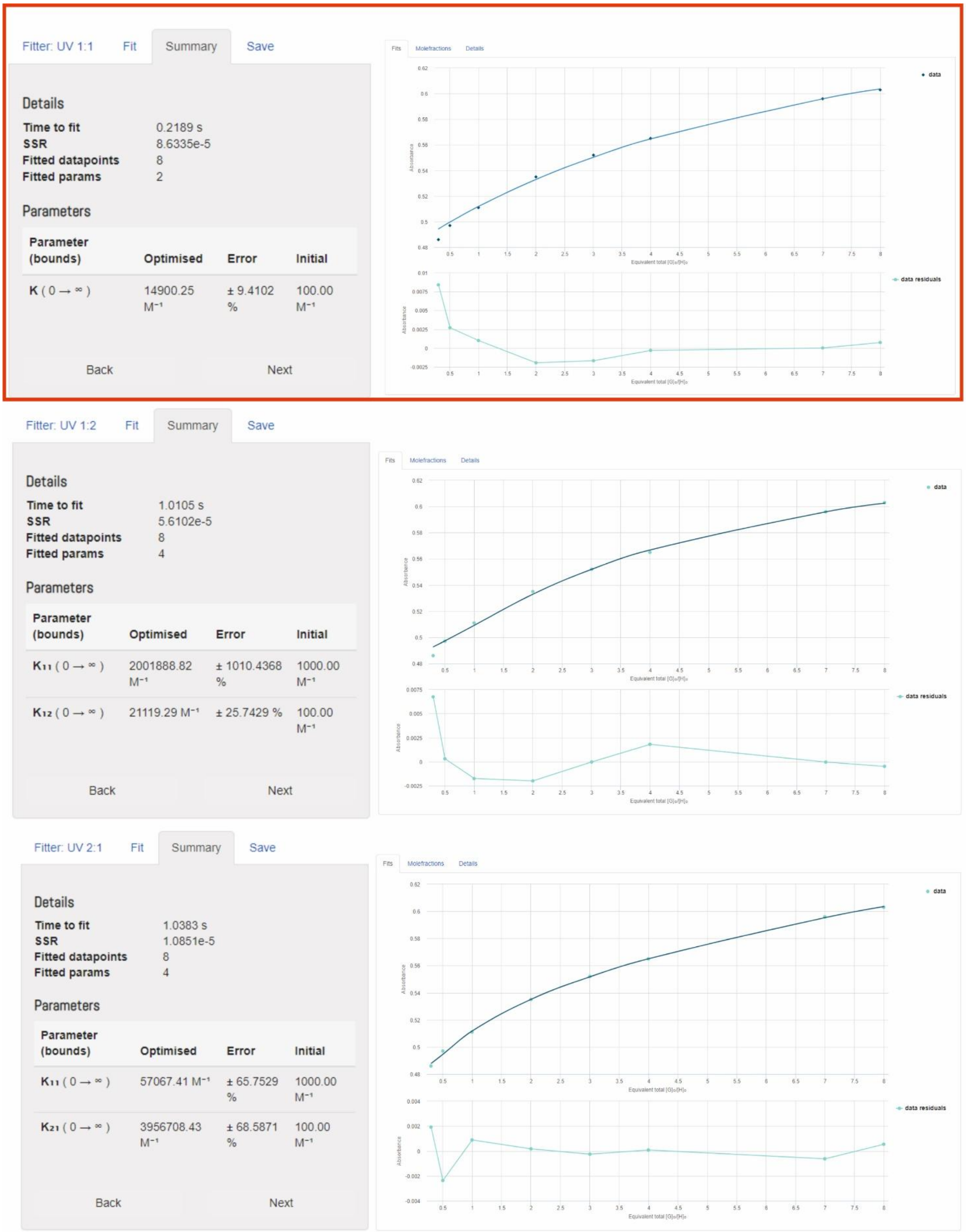

Figure S42. Bindfit (Fit data to 1:1, 1:2 and 2:1 Host-Guest equilibria) Screenshots taken from the summary window of the website supramolecular.org. This screenshots shows the raw data for UV-vis titration of **5** with **3b**.

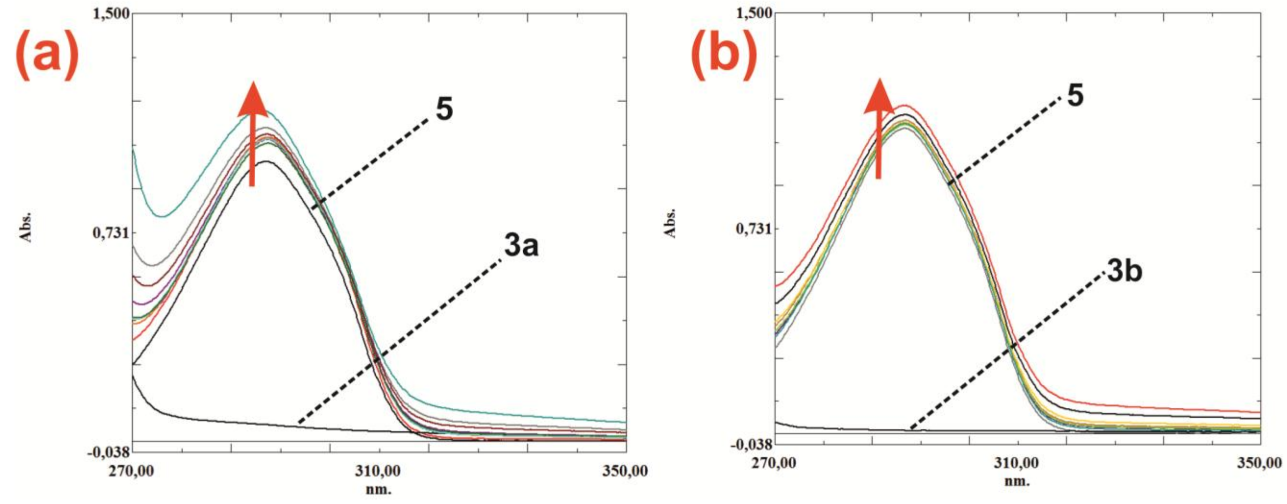

Figure S43. UV-vis titration spectra for the system: (a) pillar[5]arene **5** (1×10^{-5}M)/ **3a** ($C = 3.33\times10^{-6} - 5\times10^{-5}$ M) in buffer (pH=7.4); (b) pillar[5]arene **5** (1×10^{-5} M)/ **3b** ($C = 3.33 \times 10^{-6} - 5 \times 10^{-5}$ M) in buffer (pH=7.4).
